# BET inhibition induces GDH1-dependent glutamine metabolic remodeling and vulnerability in liver cancer

**DOI:** 10.1093/lifemeta/loae016

**Published:** 2024-04-26

**Authors:** Wen Mi, Jianwei You, Liucheng Li, Lingzhi Zhu, Xinyi Xia, Li Yang, Fei Li, Yi Xu, Junfeng Bi, Pingyu Liu, Li Chen, Fuming Li

**Affiliations:** Shanghai Key Laboratory of Metabolic Remodeling and Health, Institute of Metabolism and Integrative Biology, Fudan University, Shanghai 200438, China; Shanghai Key Laboratory of Metabolic Remodeling and Health, Institute of Metabolism and Integrative Biology, Fudan University, Shanghai 200438, China; Shanghai Key Laboratory of Metabolic Remodeling and Health, Institute of Metabolism and Integrative Biology, Fudan University, Shanghai 200438, China; Shanghai Key Laboratory of Metabolic Remodeling and Health, Institute of Metabolism and Integrative Biology, Fudan University, Shanghai 200438, China; Shanghai Key Laboratory of Metabolic Remodeling and Health, Institute of Metabolism and Integrative Biology, Fudan University, Shanghai 200438, China; Human Phenome Institute, Zhangjiang Fudan International Innovation Center, Fudan University, Shanghai 201203, China; Department of Pathology, School of Basic Medical Sciences, Fudan University, Shanghai 200032, China; Frontier Innovation Center, School of Basic Medical Sciences, Fudan University, Shanghai 200032, China; Shanghai Key Laboratory of Metabolic Remodeling and Health, Institute of Metabolism and Integrative Biology, Fudan University, Shanghai 200438, China; Shanghai Key Laboratory of Metabolic Remodeling and Health, Institute of Metabolism and Integrative Biology, Fudan University, Shanghai 200438, China; Human Phenome Institute, Zhangjiang Fudan International Innovation Center, Fudan University, Shanghai 201203, China; Shanghai Key Laboratory of Metabolic Remodeling and Health, Institute of Metabolism and Integrative Biology, Fudan University, Shanghai 200438, China; Shanghai Key Laboratory of Metabolic Remodeling and Health, Institute of Metabolism and Integrative Biology, Fudan University, Shanghai 200438, China

**Keywords:** BET, glutamate dehydrogenase 1, oxidative phosphorylation, glutamine metabolism, synthetic lethality

## Abstract

Bromodomain and extra-terminal domain (BET) proteins, which function partly through MYC proto-oncogene (MYC), are critical epigenetic readers and emerging therapeutic targets in cancer. Whether and how BET inhibition simultaneously induces metabolic remodeling in cancer cells remains unclear. Here we find that even transient BET inhibition by JQ-1 and other pan-BET inhibitors (pan-BETis) blunts liver cancer cell proliferation and tumor growth. BET inhibition decreases glycolytic gene expression but enhances mitochondrial glucose and glutamine oxidative metabolism revealed by metabolomics and isotope labeling analysis. Specifically, BET inhibition downregulates *miR-30a* to upregulate glutamate dehydrogenase 1 (GDH1) independent of MYC, which produces α-ketoglutarate for mitochondrial oxidative phosphorylation (OXPHOS). Targeting GDH1 or OXPHOS is synthetic lethal to BET inhibition, and combined BET and OXPHOS inhibition therapeutically prevents liver tumor growth *in vitro* and *in vivo*. Together, we uncover an important epigenetic-metabolic crosstalk whereby BET inhibition induces MYC-independent and GDH1-dependent glutamine metabolic remodeling that can be exploited for innovative combination therapy of liver cancer.

## Introduction

Primary hepatocellular carcinoma (HCC), the major type of liver cancer, is a leading cause of worldwide cancer-related death [1–3]. End-stage liver cancer patients have limited treatment options due to the lack of druggable targets, and the treatment outcome is usually complicated by the heterogeneous tumor microenvironment [4]. Recent clinical trials showed that only a small portion of patients initially respond to either molecular targeted therapy or combined anti-angiogenic therapy and immune checkpoint blockade, but eventually develop drug resistance [5, 6]. Therefore, it’s urgent to understand the drug resistance mechanisms and develop improved intervention strategies for liver cancer treatment.

One hallmark of cancer is deregulated metabolism, which contributes to tumor progression, metastasis, and relapse through cancer cell-intrinsic metabolic remodeling and metabolic interactions within the tumor microenvironment [7–10]. HCC develops from transformed hepatocytes with features of metabolic remodeling [11]. For example, aberrant Wnt signaling upregulates glutamine synthetase (GLUL) to increase glutamine levels and activate the mammalian target of rapamycin (mTOR) signaling pathway to facilitate tumor progression [12], and loss of p53 activates sterol regulatory element binding transcription factor 2 (SREBP-2)-driven mevalonate pathway to promote liver tumorigenesis [13]. Moreover, loss of urea cycle enzymes in HCC renders cancer cells auxotrophic for arginine [14], and argininosuccinate synthase 1 (ASS1) downregulation enables cancer cells to accumulate aspartate for pyrimidine synthesis necessary for cell proliferation [15]. Recently, we demonstrated that gluconeogenic gene fructose-1,6-bisphosphatase 1 (*FBP1*) is universally silenced during liver tumorigenesis, and hepatic FBP1 loss in mice disrupts liver metabolism and promotes liver tumor progression, through a hepatic stellate cell senescence secretome and extracellular vesicle-mediated communication between hepatocytes and natural killer cells [16, 17]. While these studies have identified different metabolic enzymes and pathways modulating liver tumor initiation and progression, how liver cancer cells undergo metabolic adaptations in response to specific drug treatment has not been fully explored.

Bromodomain and extra-terminal domain (BET) proteins, which comprise bromodomain-containing protein 2 (BRD2), BRD3, and BRD4, and the testis-restricted BRDT (bromodomain testis-specific protein), are epigenetic readers containing two tandem bromodomains (BD1 and BD2), an extra-terminal domain (ET), and a C-terminal domain (CTD) [18]. By recognizing acetylated lysine of histone and non-histone proteins, BET proteins act as scaffolds to recruit many other proteins to promoters and enhancers (especially at the super-enhancers) of target genes and to facilitate the transcription. Notable BET protein target genes include *MYC* proto-oncogene (*MYC*)*,* B cell lymphoma 2 (*BCL2*)*, BCL6,* cyclin-dependent kinase 4 (*CDK4*)*,* and *CDK6*, which are involved in cell cycle progression, cell survival, and other biological processes. Among these, MYC is also a master regulator of cellular metabolism. Overexpressed MYC, observed in several types of cancer, activates target genes of glycolysis, mitochondrial biogenesis/oxidative phosphorylation (OXPHOS), glutamine metabolism, *de novo* nucleotide synthesis, and many other metabolic pathways [19, 20]. Given the overexpression of BET proteins in cancer, previous studies have shown the promise of targeting BET proteins as an intervention strategy in hematologic malignancies, breast cancer [21], prostate cancer [22], etc. Consistently, one study showed that the pan-BET inhibitor (pan-BETi) JQ-1 reduces liver fibrosis [23], or liver tumor burden in a mouse model of non-alcoholic steatohepatitis (NASH)-HCC [24]. In the meanwhile, different classes of pan-BETis are now in clinical trials, which confirm their antitumor potential [25, 26]. However, the efficacy of BET inhibition as monotherapy is limited, making it necessary to develop combination therapies with other anticancer agents, currently including kinase inhibitors, epigenetic drugs, immune modulators, and hormone therapy [26]. Since cancer cells dynamically undergo metabolic remodeling during drug treatment, how BET inhibition induces MYC-dependent or independent metabolic adaptations in cancer cells remains unclear. Addressing this question will add more mechanistic insights into how epigenetic-metabolic crosstalk contributes to tumor growth, and more importantly, uncover metabolic vulnerabilities that could be exploited to enhance the therapeutic efficacy of BET inhibition in cancer.

In this study, we show that BET inhibition induces MYC-independent and glutamate dehydrogenase 1 (GDH1)-dependent glutamine metabolic remodeling in liver cancer. Targeting the GDH1-OXPHOS metabolic axis is “synthetic lethal” to BET inhibition, which limits liver tumor progression in different mouse models. Our study thus provides an innovative combination therapy for liver cancer treatment.

## Results

### Transient BET inhibition blunts HCC cell growth and induces differentiation

**Figure 1:**
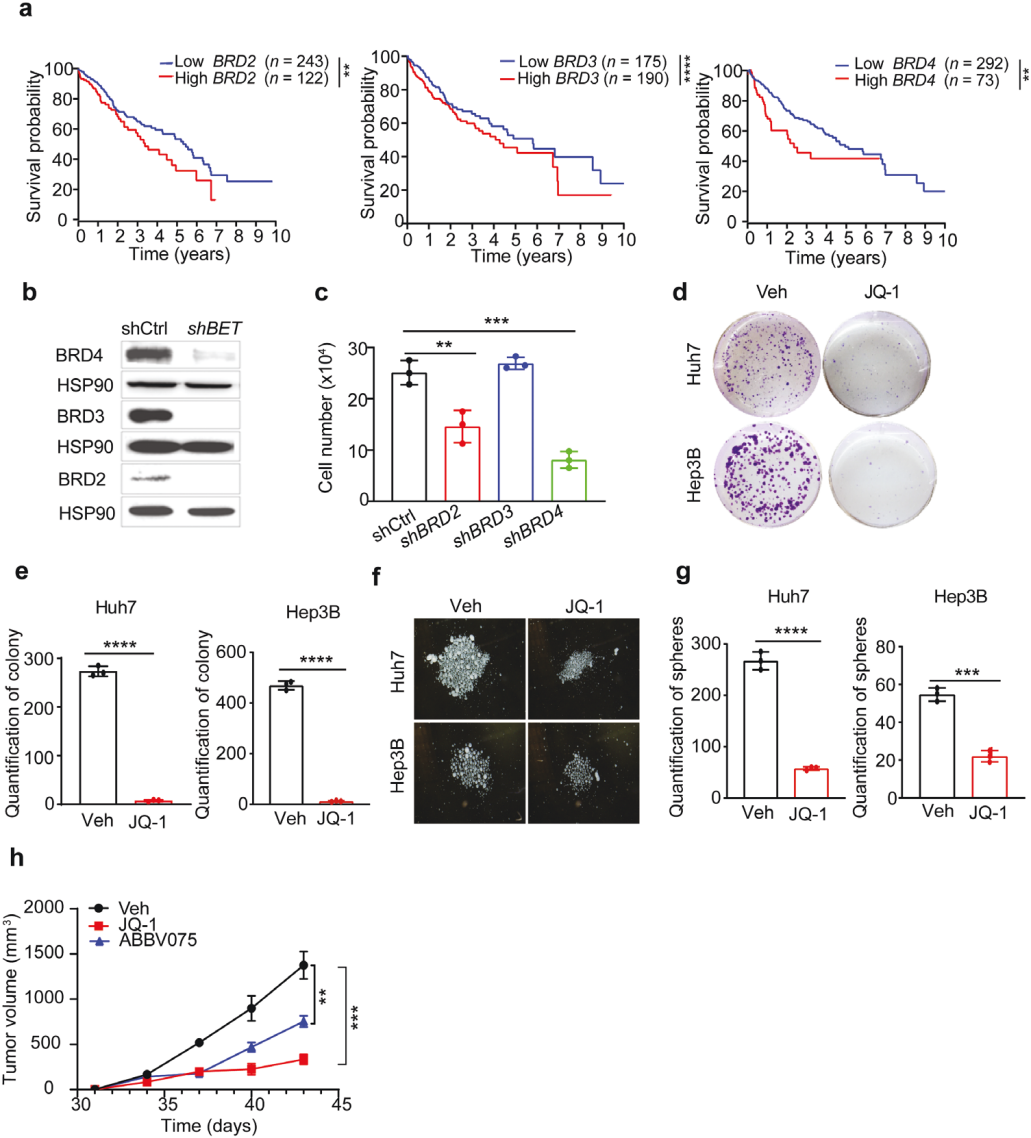
BET inhibition blunts HCC cell growth and induces differentiation. (a) Kaplan–Meier overall survival plots stratified by *BRD2, BRD3*, and *BRD4* mRNA levels from TCGA HCC database. (b) Western blot analysis of indicated proteins from control and *BET*-knockdown Huh7 cells. (c) Cell number quantification of control and *BET*-knockdown Huh7 cells after growing for 72 h. (d) Representative clonogenicity assays from replated Huh7 and Hep3B cells after pretreatment with vehicle or JQ-1 (0.5 μmol/L) for 48 h. (e) Colony number quantification of replated Huh7 and Hep3B cells after pretreatment with vehicle or JQ-1 (0.5 μmol/L) for 48 h. (f and g) Representative Huh7 and Hep3B sphere cultures (f) and quantification (g) from the vehicle and JQ-1 pretreatment groups. (h) Huh7 xenograft tumor growth curves from vehicle (*n* = 4), JQ-1 (*n* = 6), or ABBV075 (*n* = 6) pretreatment groups. All statistical graphs except that in a show the mean ± SEM. *P* values were calculated using a log-rank Mantel–Cox test (a), one-way ANOVA (c and h), and a two-tailed Student’s *t*-test (e and g). All experiments were performed in biological triplicate. ^*^P < 0.05, ^**^P < 0.01, ^***^P < 0.001, ^****^P < 0.0001.

Among the BET family, BRD4 is the best-studied member to regulate tumorigenesis through cancer cell autonomous and non-autonomous mechanisms [18, 26]. We set out to determine the correlation of individual BET gene expression with liver cancer patient survival. Analysis of The Cancer Genome Atlas (TCGA) dataset demonstrated that elevated *BRD2*, *BRD3*, and *BRD4* transcript levels were negatively correlated with patient survival (Fig. 1a). Meanwhile, short hairpin RNA (shRNA)-mediated knockdown of *BRD2* and *BRD4* in the liver cancer cell line Huh7 significantly slowed down cell proliferation, suggesting that both BRD2 and BRD4 were important for liver cancer cell growth (Fig. 1b and c). To pharmacologically target BET proteins, we applied the well-established pan-BETi JQ-1 [27] to treat Huh7 and Hep3B cells, two most sensitive cell lines according to the “The Genomics of Drug Sensitivity in Cancer Project” (Supplementary Fig. S1a). JQ-1 dose-dependently decreased growth without inducing dramatic death in both cell lines. Similarly, another orally bioavailable and potent pan-BETi ABBV075 [28] also blunted Huh7 and Hep3B cell growth in a dose-dependent manner (Supplementary Fig. S1b−d). Notably, even after transient (48 h) JQ-1 exposure, both Huh7 and Hep3B cells exhibited much slower proliferation in cell growth assay (Supplementary Fig. S1e), and formed fewer colonies in clonogenicity assay compared to vehicle-treated cells (Fig. 1d and e). In a serum-free suspension culture condition to enrich cancer stem cell (CSC)-derived spheres [29], transient JQ-1 pre-treated Huh7 and Hep3B cells gave rise to much smaller and fewer spheres, further supporting reduced tumorigenicity potential (Fig. 1f and g). Accordingly, when control and BETi-exposed Huh7 cells were subcutaneously transplanted to immune-compromised mice, the resultant xenograft tumor growth was profoundly dampened, as supported by decreased tumor volume change (Fig. 1h).

BET proteins contain two bromodomains, and it has been shown that BD1-specific inhibitors phenocopy the effects of pan-BETis whereas BD2 inhibitors are predominantly effective in inflammatory and autoimmune disease models [30]. We treated Huh7 cells with BD1 inhibitor (GSK778) or BD2 inhibitor (GDK046) and found that Huh7 cells were only sensitive to BD1 inhibitor but refractory to BD2 inhibitor (Supplementary Fig. S1f), suggesting that BD1 mediated the oncogenic functions of BET proteins in liver cancer. Collectively, these results indicated that even transient BET inhibition is sufficient to blunt liver cancer cell proliferation and tumor growth.

### BET inhibition alters glucose and glutamine metabolic gene expression

**Figure 2:**
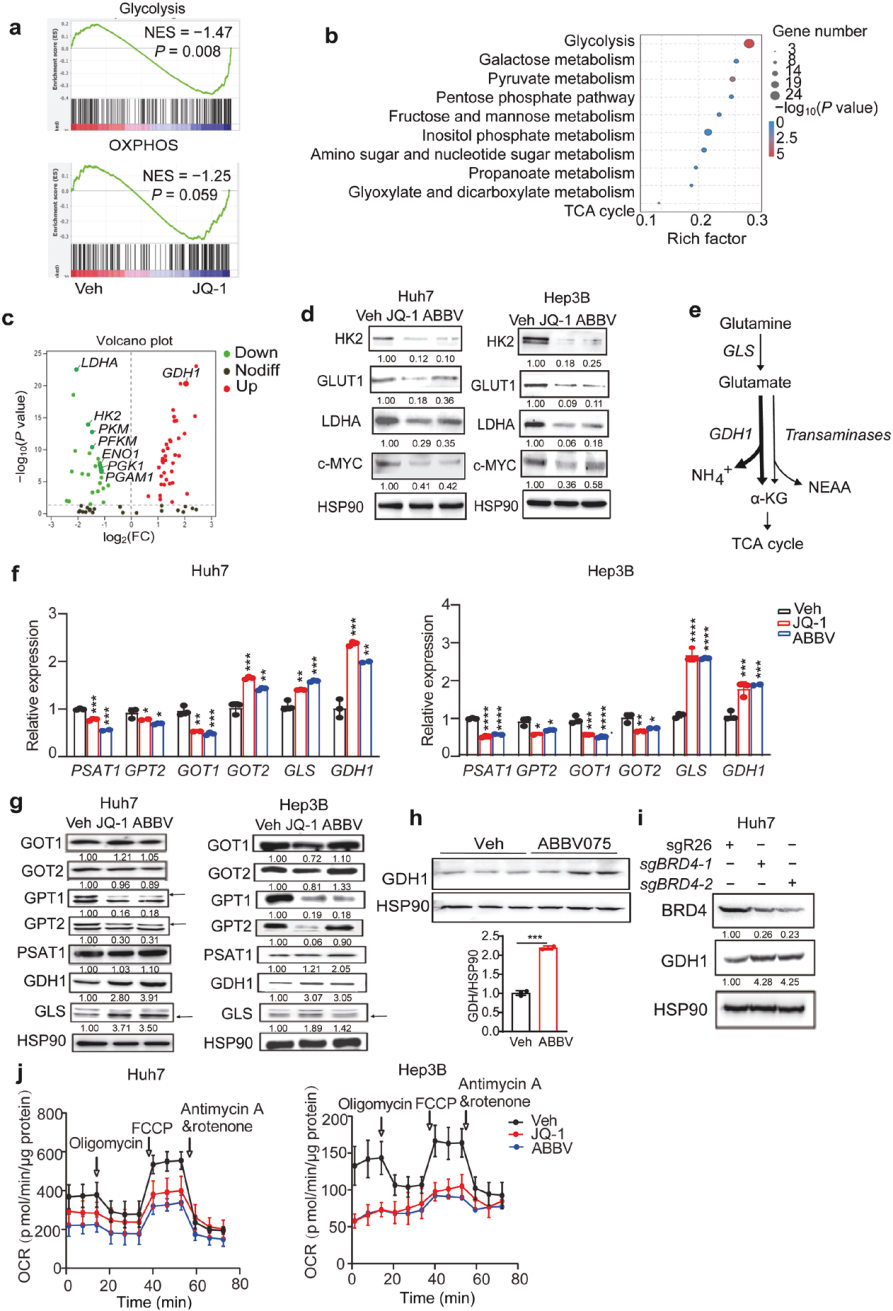
BET inhibition alters glucose and glutamine metabolic gene expression. (a) GSEA for glycolysis and OXPHOS from the vehicle and JQ-1 groups based on RNA-seq data. (b) Top-ranked KEGG metabolic pathways from the vehicle and JQ-1 groups based on RNA-seq data. (c) A volcano plot of indicated metabolic gene expression based on RNA-seq data. (d) Western blot analysis of glycolytic enzymes from the vehicle and BETi-treated Huh7 and Hep3B cell lysates. HSP90 is used as a loading control. (e) The scheme of glutaminolysis. (f and g) qPCR (f) and western blot (g) analysis of glutamine metabolic gene expression in BETi-treated Huh7 and Hep3B cells. (h) Western blot analysis and quantification of GDH1 expression in Huh7 xenograft tumor model using ABBV075. *n* = 3 tumors for each group. (i) Western blot analysis of GDH1 from sgCtrl and *sgBRD4* Huh7 cell lysates. (j) Seahorse assays of the vehicle and BETi-treated Huh7 and Hep3B cells. All statistical graphs show the mean ± SEM. *P* values were calculated using one-way ANOVA. All experiments were performed in biological triplicate. ^*^P < 0.05, ^**^P < 0.01, ^***^P < 0.001, ^****^P < 0.0001.

To address whether BET inhibition results in metabolic remodeling in liver cancer cells, RNA sequencing (RNA-seq) was performed to compare the transcriptome and metabolic gene expression in control and transient JQ-1-treated Huh7 cells. This profiling uncovered significantly altered mRNAs (*P*adj < 0.05, more than 2-fold change), including 1743 genes upregulated and 2027 genes downregulated. Gene set enrichment analysis (GSEA) further uncovered top-ranked gene sets of glycolysis, OXPHOS, purine metabolism, and pyrimidine metabolism that were significantly altered by JQ-1 exposure (Fig. 2a; Supplementary Fig. S2a). Consistently, Kyoto Encyclopedia of Genes and Genomes (KEGG) analysis of metabolic pathways identified glycolysis as one of the top-ranked pathways with distinct gene expression patterns (Fig. 2b). Indeed, a volcano plot of metabolic gene expressions clearly showed that many glycolytic gene transcripts were downregulated (Fig. 2c; Suppplementary Fig. S2b), and both mRNA and protein levels of representative glycolytic genes (glucose transporter 1 (*GLUT1*), hexokinase 2 (*HK2*), and lactate dehydrogenase A (*LDHA*)) were validated by quantitative reverse-PCR (qPCR) and western blot analysis, respectively (Fig. 2d; Supplementary Fig. S2c).

Glutaminolysis provides the cells with a nitrogen source, non-essential amino acids (NEAA) including alanine, aspartate, and proline, as well as α-ketoglutarate (α-KG) which can replenish the tricarboxylic acid (TCA) cycle [31]. Specifically, mitochondrial glutamine is converted to glutamate by glutaminase 1 (GLS1), and glutamate is then catabolized either through GDH1 to produce α-KG or through different transaminases to generate NEAA and α-KG (Fig. 2e). Since RNA-seq analysis revealed *GDH1* upregulation by JQ-1 treatment in Huh7 cells, we then experimentally compared glutaminolysis-related gene expressions. Both mRNA and protein levels of GLS1 and GDH1 were consistently elevated by JQ-1 or ABBV075 treatment in Huh7 and Hep3B cells while transaminase-coding genes exhibited heterogeneous changes in mRNA or protein levels. For example, glutamate pyruvate transaminase 1 (*GPT1*) and *GPT2* expression levels were decreased, while phosphoserine aminotransferase 1 (*PSAT1*), glutamic-oxaloacetic transaminase 1 (*GOT1*), and *GOT2* levels remained largely unchanged (Fig. 2f and g). Similarly, GDH1 upregulation was also detected in ABBV075-treated Huh7 xenografts (Fig. 2h), as well as Huh7 cells with CRISPR-mediated BRD4 depletion (Fig. 2i).

In addition to glycolysis and glutaminolysis genes, we also wondered whether BET inhibition may directly impact mitochondrial gene expression. Protein levels of mitochondrial transcription factor A (TFAM), a critical transcriptional regulator of mitochondrial biogenesis, were reduced by BETi treatment (Supplementary Fig. S2d). Consistently, TFAM targets encoding mitochondrial electron transport chain subunits (*MT-ND1*, *NDUFA11*, *ATP5D*, *ATP8*, *UCP2,* and *COX17*) exhibited reduced transcription by BETi treatment (Supplementary Fig. S2e). Importantly, BETi-treated Huh7 and Hep3B cells had reduced oxygen consumption rate (OCR) in seahorse assays, accompanied by decreased basal and maximal respiration, as well as ATP production, indicating defective mitochondrial function (Fig. 2j; Supplementary Fig. S2f). Together, we concluded that BET inhibition potentially elicits metabolic remodeling in liver cancer cells.

### BET inhibition enhances glutamine-derived TCA anaplerosis and shifts energy supply to TCA cycle

**Figure 3:**
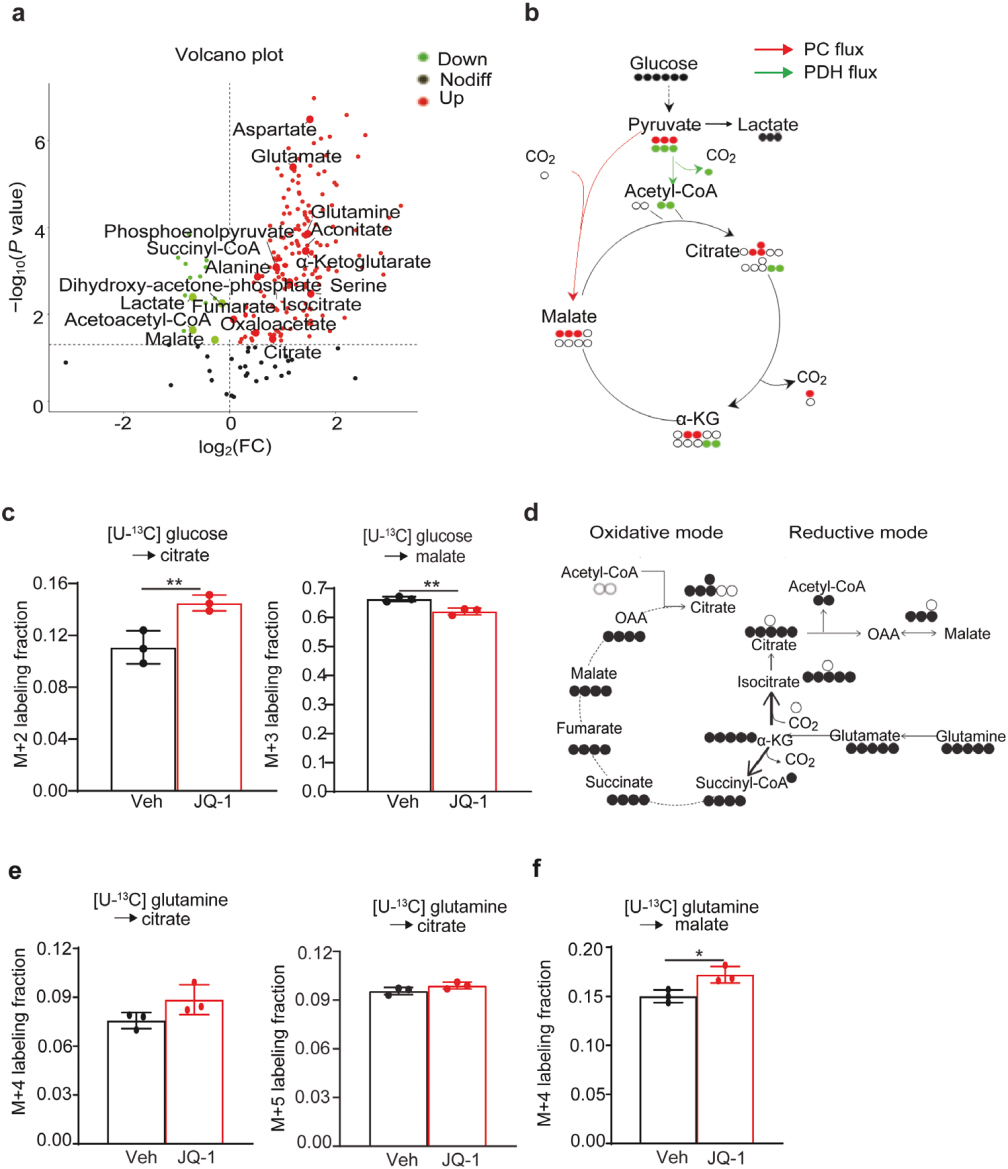
BET inhibition induces glutamine metabolic remodeling. (a) A volcano plot of indicated metabolite abundance from targeted metabolomics of vehicle and JQ-1-treated Huh7 cells. (b) A scheme of glucose labeling followed by PC or PDH-dependent metabolism to synthesize citrate and malate. (c) Mass isotopologue analysis of M+2 citrate and M+3 malate in the vehicle and JQ-1 pre-treated Huh7 cells cultured with U-^13^C glucose. (d) A scheme of glutamine labeling followed by oxidative or reductive metabolism to synthesize citrate. (e) Mass isotopologue analysis of M+5 and M+4 citrate in the vehicle and JQ-1 pre-treated Huh7 cells cultured with U-^13^C glutamine. (f) Mass isotopologue analysis of M+4 malate in the vehicle and JQ-1 pre-treated Huh7 cells cultured with U-^13^C glutamine. All statistical graphs show the mean ± SEM. *P* values were calculated using a two-tailed Student’s *t*-test. All experiments were performed in biological triplicate. ^*^P < 0.05, ^**^P < 0.01.

To profile the metabolic remodeling process upon BET inhibition, we performed metabolomics analysis on vehicle and JQ-1-treated Huh7 cells. This profiling uncovered top-ranked and enriched metabolic pathways, including glycolysis, glutamine/glutamate metabolism, glutathione metabolism, taurine and hypotaurine metabolism, and others (Supplementary Fig. S3a and b). In line with the elevated GLS1 and GDH1 expression, the abundance of glutaminolysis and TCA cycle intermediate metabolites (glutamate, α-KG, aspartate, succinyl-coenzyme A (succinyl-CoA), and isocitrate) was increased upon JQ-1 treatment, indicating enhanced glutamine anaplerosis for TCA cycle (Fig. 3a). Notably, phosphorylation of the energy sensor adenosine monophosphate (AMP)-activated protein kinase (pAMPK) remained unchanged by JQ-1 treatment, collectively suggesting that BETi-exposed cells can maintain energy homeostasis despite impaired growth (Supplementary Fig. S3c).

To further validate the above results and determine the effects of BETi on glucose and glutamine metabolic remodeling, we sought to measure relevant metabolic fluxes using isotope tracing techniques in the vehicle and JQ-1-treated Huh7 cells. Specifically, we cultured cells with uniformly labeled glucose ([U-^13^C] glucose) and measured ^13^C enrichment of intracellular metabolites by mass spectrometry. [U-^13^C] glucose-derived M+3 pyruvate (containing 3 ^13^C carbons) undergoes oxidative decarboxylation to generate [1,2-^13^C] acetyl-CoA and subsequently M+2 labeled citrate (Fig. 3b). We observed that the pyruvate decarboxylation flux increased (Fig. 3c), likely to compensate for the energy supply loss due to the reduced glycolysis activity. Meanwhile, pyruvate could also enter the TCA cycle via pyruvate carboxylase-mediated carboxylation flux to supply TCA substrate (Fig. 3b). The flux modestly decreased as demonstrated by decreased M+3 malate (Fig. 3c).

We also cultured cells with uniformly labeled glutamine ([U-^13^C] glutamine) to probe glutamine-related metabolic fluxes. [U-^13^C] glutamine-derived α-KG enters the TCA cycle and undergoes oxidative metabolism (oxidative mode indicating energy supply) where TCA cycle metabolites including malate and citrate are M+4 labeled. Alternatively, α-KG undergoes reductive carboxylation (reductive mode indicating reductive biosynthesis) where α-KG is converted to M+5 isocitrate and finally to M+5 citrate (Fig. 3d). Notably, as a function of the increased α-KG to citrate ratio [32], reductive glutamine metabolism is essential to support growth of tumor cells with mitochondrial defects or maintain redox homeostasis during anchorage-independent growth [33–36]. Flux analysis revealed that JQ-1 exposure resulted in increased labeling fraction of citrate M+4 and malate M+4 to different extents, while the labeling fraction of citrate M+5 remained comparable (Fig. 3e and f). This labeling resulted in enhanced oxidative glutamine metabolism, while reductive glutamine metabolism was minimally affected, and it was consistent with higher *GLS* and *GDH1* transcript levels upon BET inhibition.

Together, both metabolomics profiling and isotope labeling results suggest that BET inhibition induces glutamine-derived TCA anaplerosis and energy supply shift to TCA cycle to support cell growth.

### GDH1-dependent glutamine metabolism maintains cancer cell viability after BET inhibition

**Figure 4:**
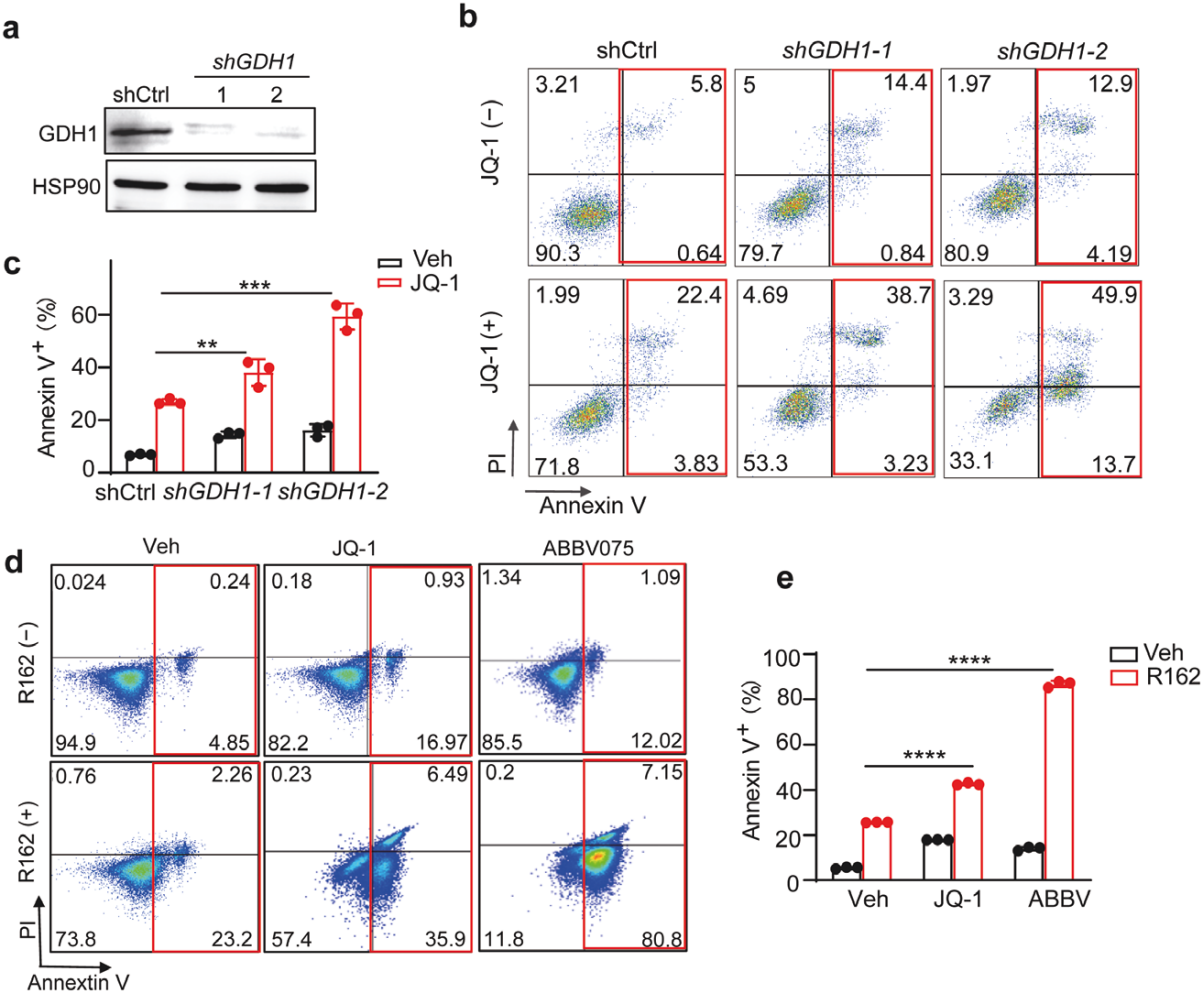
*GDH1*-dependent glutamine metabolism maintains cell viability after BET inhibition. (a) Western blot analysis of *GDH1* expression in control (shCtrl) and *GDH1* knockdown (*shGDH1*) Huh7 cells. HSP90 is used as a loading control. (b) Representative flow cytometry plot of PI/Annexin V staining of Huh7 cells from indicated groups. (c) Statistic analysis of the percentage of Annexin V^+^ cells from indicated groups in b. (d) Representative flow cytometry plot of PI/Annexin V staining in the vehicle and JQ-1/R162 treated Huh7 cells. (e) Statistic analysis of the percentage of annexin V^+^ cells from indicated groups in d. All statistical graphs show the mean ± SEM. *P* values were calculated using one-way ANOVA. All experiments were performed in biological triplicate. ^****^P < 0.0001.

We have now shown that BET inhibition upregulated GDH1 expression and simultaneously decreased certain mitochondrial transaminase (GPT2) expression, whereas the abundance of α-KG, a common product of these enzymes, was not reduced but increased instead. We thus reasoned that GDH1 upregulation preserved α-KG levels for TCA cycle to maintain energy metabolism. If this is the case, GDH1 would be required for cell growth and/or viability after BET inhibition. To test this, we knocked down *GDH1* in Huh7 cells, and then treated control (shCtrl) and *GDH1* knockdown (*shGDH1*) cells with JQ-1 (Fig. 4a). As shown in Fig. 4b and c, compared to control cells, *GDH1*-deficient Huh7 cells were extremely sensitive to JQ-1 treatment and dramatic cell death was detected by propidium iodide (PI)/Annexin V staining and flow cytometry.

It was previously shown that a small molecule inhibitor R162 can abrogate GDH1 enzyme activity and dramatically decrease intracellular α-KG levels in lung and breast cancer cells [37]. We then investigated whether R162-treated Huh7 cells would similarly be vulnerable to BETi. Although R162 itself did not induce profound cell death in Huh7 cells, combined R162 and JQ-1 or ABBV075 exposure remarkably induced apoptosis (Fig. 4d and e). As expected, pharmacologically targeting GLS1 using CB839 [38], which acts upstream of GDH1, also sensitized Huh7 cells to JQ-1 treatment (Supplementary Fig. S4a–c). Taken together, these results support the conclusion that BET inhibition induces GDH1 dependence in liver cancer cells, and GDH1 enzymatic activity is required for the viability of liver cancer cells upon BET inhibition.

### BET inhibition induces GDH1 expression through *miR-30a* downregulation

**Figure 5:**
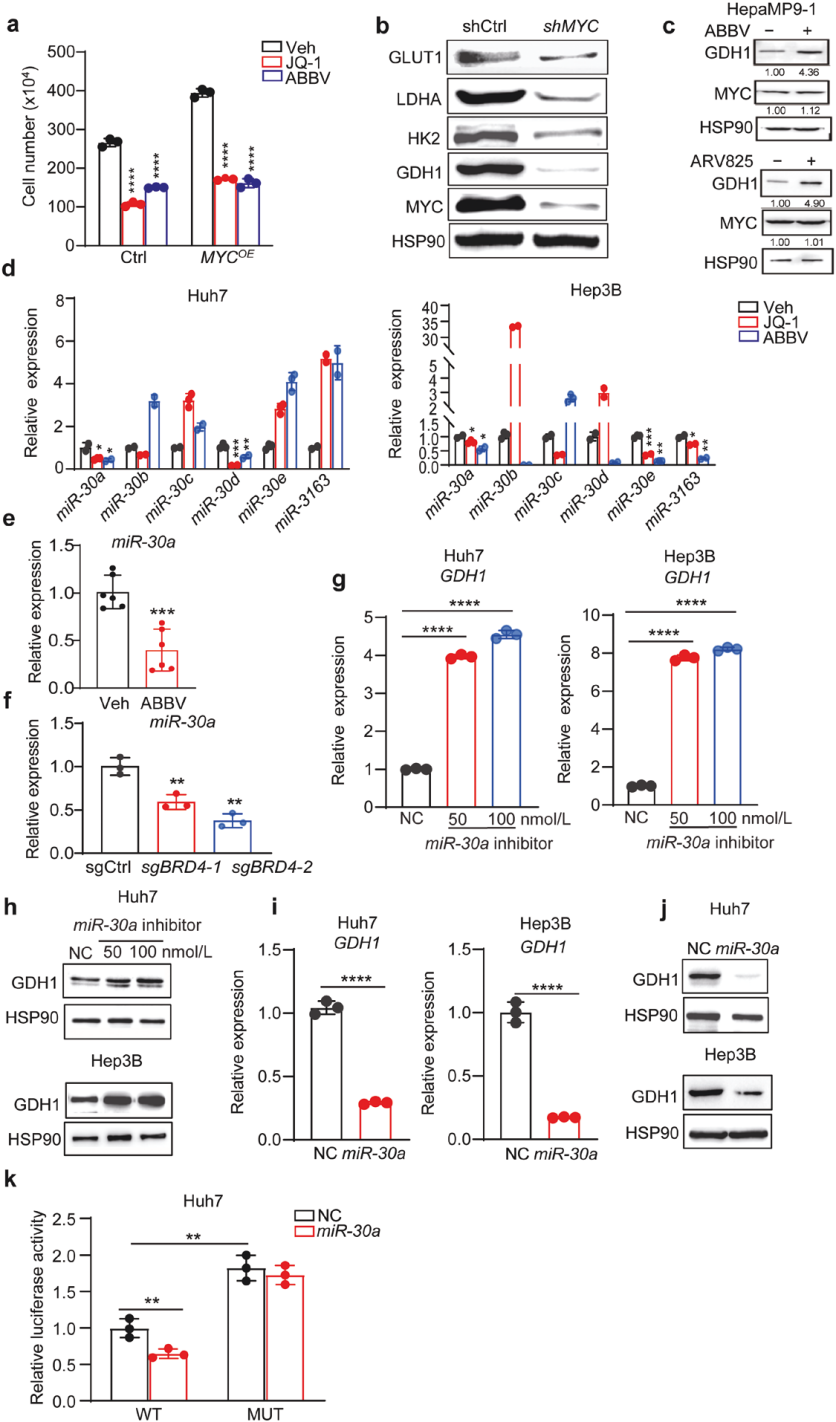
BET inhibition induces *GDH1* expression through *miR-30a* downregulation. (a) Quantification of cell numbers in control and *MYC*-overexpressed Huh7 cells treated with BETis. (b) Western blot analysis of indicated proteins on control and *MYC* knockdown Huh7 cells. HSP90 is used as a loading control. (c) Western blot analysis of GDH1 and MYC expression in HepaMP9-1 cells treated with ABBV075 (5 nmol/L) and ARV825 (5 nmol/L) for 48 h. HSP90 serves as a loading control. (d) qPCR analysis of indicated miR30 family genes in vehicle and BETi-treated Huh7 and Hep3B cells. (e) qPCR analysis of *miR-30a* levels in vehicle (*n* = 5) and ABBV075 (*n* = 6) treated Huh7 xenografts. (f) qPCR analysis of *miR-30a* expression from sgCtrl and *sgBRD4* Huh7 cell lysates. (g) qPCR analysis of *GDH1* mRNA levels in Huh7 and Hep3B cells transfected with control or *miR-30a* inhibitor at indicated doses. (h) Western blot analysis of *GDH1* expression in Huh7 and Hep3B cells transfected with control and *miR-30a* inhibitor at indicated doses. (i) qPCR analysis of *GDH1* mRNA levels in control and *miR30a*-overexpressed Huh7 and Hep3B cells. (j) Western blot analysis of *GDH1* expression in control and *miR30a*-overexpressed Huh7 and Hep3B cells. (k) Luciferase assay in Huh7 cells transfected with indicated reporter, control, and expression plasmids. All statistical graphs show the mean ± SEM. *P* values were calculated using one-way ANOVA (a, d, g, f, and k) and a two-tailed Student’s *t*-test (e and i). All experiments were performed in biological triplicate. ^*^P < 0.05, ^**^P < 0.01, ^***^P < 0.001, ^****^P < 0.0001.

We next addressed how BET inhibition upregulated GDH1 expression in liver cancer cells. MYC, a master regulator of cellular metabolism, is the best-known BET protein target and mediates several oncogenic functions of BET proteins in cancer. Targeting BET proteins has been considered an actionable way to decrease MYC expression [27, 39]. We noted that BETi decreased MYC levels in liver cancer cells, and MYC targets were identified as the top-ranked gene sets with altered expression by BETi (Fig. 2d; Supplementary Fig. S5a). We thus wondered whether the functional effects of BET inhibition were associated with MYC expression. Unexpectedly, Huh7 cells with *MYC* overexpression were equally sensitive to JQ-1 or ABBV075, as both BETis comparably decreased cell growth in parental and *MYC*-overexpressed cells (Fig. 5a; Supplementary Fig. S5b). Different from BETis, *MYC* knockdown did not increase but decreased GDH1 expression, while other targets (GLUT1, HK2, and LDHA) were downregulated similarly to BET inhibition (Fig. 5b). Growth of HepaMP9-1 cells, a murine HCC cell line driven by constitutive *MYC* overexpression and p53 loss (*MYC*^*OE*^*; Trp53*^*KO*^), was also impaired by dose-dependent JQ-1 or ABBV075 treatment (Supplementary Fig. S5c). GDH1 was consistently upregulated in HepaMP9-1 cells treated by ABBV075 or ARV-825, a BRD4 proteolysis-targeting chimera (PROTAC) (Fig. 5c). Importantly, neither ABBV075 nor ARV-825 altered MYC expression in HepaMP9-1 cells, collectively suggesting that BET inhibition upregulated GDH1 expression largely through MYC-independent mechanisms.

Increased mRNA levels result from either transcriptional activation and/or post-transcriptional regulation such as elevated mRNA stability. Since BET proteins generally facilitate gene transcription, one would predict that BETi will inhibit gene transcription. Associated with elevated *GDH1* mRNA levels, we found a nearly 2-fold increase in the mRNA stability after JQ-1 exposure (Supplementary Fig. S5d). Therefore, we explored post-transcriptional regulation of *GDH1* by miRNAs, which negatively regulate target mRNA stability and gene expression, although we could not exclude other potential transcriptional regulation mechanisms. Using the online tool TargetScan to predict *GDH1*-bound miRNAs, the miR30 family was identified as the only one potentially targeting *GDH1* 3’ untranslated region (3’-UTR), which was conserved across several species. Among the five family members (*miR-30a−e*), only *miR-30a-5p* (*miR-30a*) had a consistent 30%−50% decrease in expression in Huh7 and Hep3B cells by JQ-1 or ABBV075 treatment (Fig. 5d). Similarly, ABBV075 treatment reduced *miR-30a* levels in Huh7 xenografts (Fig. 5e), and *BRD4* deficiency also decreased *miR-30a* levels in Huh7 cells (Fig. 5f). We thus hypothesized that *miR-30a* was the major miRNA targeting *GDH1.* To validate this, we performed both inhibition and overexpression experiments and evaluated the effects on GDH1 expression. Transfection of inhibitor interfering with endogenous *miR-30a*’s action increased both mRNA and protein levels of GDH1 in Huh7 and Hep3B cells (Fig. 5g and h). Conversely, ectopic *miR-30a* expression decreased *GDH1* mRNA and protein levels in both lines (Fig. 5i and j; Supplementary Fig. S5e). We further mutated the *miR-30a* target sequence and fused wild-type (WT) or mutant (MUT) *GDH1* 3’-UTR to the C-terminal of the luciferase coding sequence (Supplementary Fig. S5f). Through dual luciferase assay, co-transfection of *miR-30a* decreased the relative activity of luciferase-WT UTR reporter compared to the vector control group, while such effects were abrogated in cells transfected with luciferase-MUT UTR reporter vector (Fig. 5k). These results provided substantial evidence to support the notion that *miR-30a* binds to 3’-UTR and negatively regulates *GDH1* mRNA stability. Analysis of the database finally further revealed a significant negative correlation between *miR-30a* and *GDH1* transcript levels (Supplementary Fig. S5g). In summary, we concluded that BET inhibition reduces *miR-30a* levels, which in turn increases *GDH1* mRNA stability leading to elevated protein levels.

### BET inhibition is synthetic lethal to OXPHOS blockage in liver cancer

**Figure 6:**
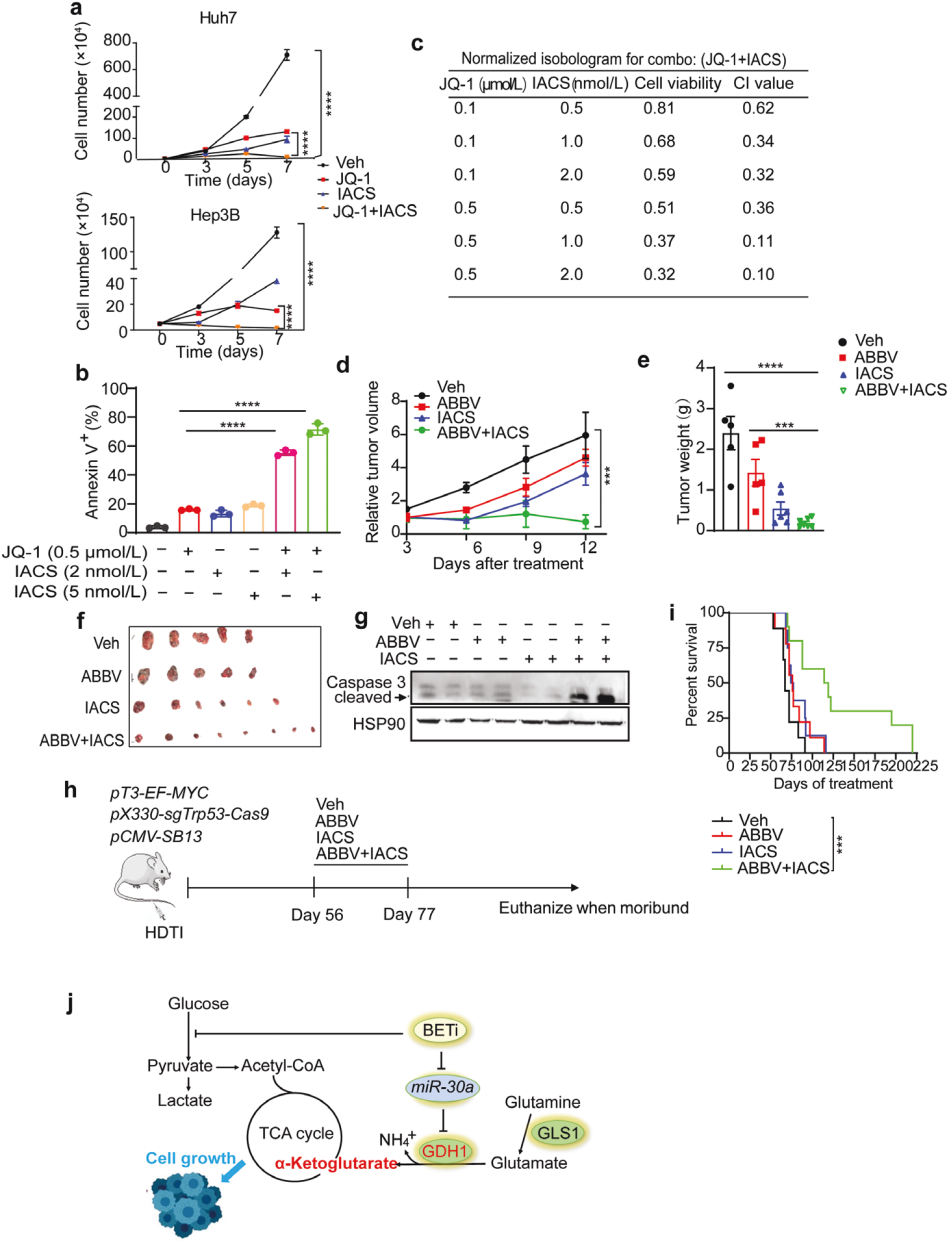
BET inhibition is synthetic lethal to OXPHOS blockage in liver cancer. (a) Huh7 and Hep3B cell growth assay from the indicated treatment groups with JQ-1 or IACS. (b) Statistic analysis of the percentage of Annexin V^+^ Huh7 cells from indicated treatment groups for 6 days. (c) Synergistic effects between JQ-1 and IACS treatment of Huh7 cells. Confidence interval (CI) values < 1 indicate synergy between two inhibitors. (d) Quantification of Huh7 xenograft tumor growth in the vehicle (*n* = 5), ABBV075 (*n* = 5), IACS (*n* = 5), and ABBV075/IACS combination (*n* = 8) treatment cohorts. (e) Quantification of Huh7 xenograft tumor weights in vehicle (*n* = 5), ABBV075 (*n* = 5), IACS (*n* = 5), and ABBV075/IACS combination (*n* = 8) treatment cohorts. (f) Representative images of Huh7 xenograft tumor in the vehicle, ABBV075, IACS, and ABBV075/IACS combination treatment cohorts. (g) Western blot analysis of cleaved caspase 3 in Huh7 xenograft tumors after combined ABBV075 and IACS exposure. (h) The scheme of liver tumorigenesis and drug treatment of *MYC*^*OE*^*; Trp53*^*ko*^ mouse model. (i) Kaplan–Meier survival curves of vehicle (*n* = 8), ABBV075 (*n* = 8), IACS (*n* = 9), and ABBV075/IACS combination (*n *= 10) treatment cohorts. All statistical graphs except that in i show the mean ± SEM. *P* values were calculated using one-way ANOVA (a, b, d, and e) and a log-rank Mantel–Cox test (i). All experiments were performed in biological triplicate. ^*^P < 0.05, ^**^P < 0.01, ^***^P < 0.001, ^****^P < 0.0001.

Because BETi-treated liver cancer cells can maintain energy homeostasis involving enhanced glutamine oxidative metabolism and anaplerosis for TCA cycle, we finally hypothesized that targeting OXPHOS would disrupt the energy balance and sensitize the cancer cells to BET inhibition. To address this, we used a potent OXPHOS inhibitor (OXPHOSi) IACS010759 (IACS) [40] to treat cancer cells and compared growth among different groups. Compared to the vehicle treatment group, either JQ-1 or IACS single treatment effectively slowed down Huh7 and Hep3B cell growth without obvious cell death, and combined JQ-1/IACS exposure more dramatically prevented cell growth and induced significant cell death (Fig. 6a and b; Supplementary Fig. S6a). JQ-1 and IACS had a strong synergistic effect, which was also evident between ABBV075 and IACS (Fig. 6c; Supplementary Fig. S6b). Combined JQ-1/IACS or ABBV075/IACS exposure significantly induced apoptosis in Huh7 cells, based on increased cleaved caspase 3 levels (Supplementary Fig. S6c). Importantly, in the mouse liver cancer cell line HepaMP9-1 driven by *MYC* overexpression and p53 loss, JQ-1/IACS co-exposure also significantly decreased the cell viability (Supplementary Fig. S6d). However, both SNU449, a liver cancer cell line much less sensitive to JQ-1 than Huh7 and Hep3B cells, and IMR90, a non-cancer cell line, did not respond to combined JQ-1/IACS exposure, as evidenced by the comparable cell number and viability among different treatment groups (Supplementary Fig. S6e and f). These results indicate that only BETi-responsive liver cancer cells were more sensitive to JQ-1/IACS combination therapy.

To validate BETi/OXPHOSi combination therapy potential *in vivo*, we first performed treatment on the Huh7 xenograft tumor model using ABBV075 and IACS. As shown in Fig. 6d, compared to the vehicle cohort, either 12-day ABBV075 or IACS monotherapy substantially blunted tumor growth, and IACS seemed to have a stronger inhibitory effect. Notably, ABBV075/IACS combination therapy more potently blunted tumor growth, and the endpoint tumor weights were much smaller than any other cohorts (Fig. 6e and f). Consistent with *in vitro* treatment, xenografts exposed to combined ABBV075/IACS exhibited much higher cleaved caspase 3 level than other groups (Fig. 6g).

We then performed drug treatment on a genetically engineered mouse model driven by *MYC* overexpression and p53 loss as previously [41]. In this model, a *MYC*-expressing transposon vector and another vector expressing Cas9 and *Trp53* sgRNA were delivered to murine hepatocytes using hydrodynamic tail vein injection (HDTI), where *MYC* overexpression and p53 loss triggered rapid liver tumorigenesis and shortened animal survival. After injection, mice randomized in different groups received three-week monotherapy or combination therapy, and their survival was monitored (Fig. 6h). Mice receiving either ABBV075 or IACS monotherapy exhibited longer survival than vehicle controls. Most importantly, the longest overall survival was observed with combined ABBV075/IACS administration (Fig. 6i). Notably, WT mice receiving combined ABBV075/IACS treatment initially lost about 10% body weight but progressively recovered after treatment, and the liver histology between the two cohorts was indistinguishable by pathological examination (Supplementary Fig. S6g and h). Based on the above results, we concluded that BETi combined with OXPHOSi represents an enhanced therapeutic option in liver cancer.

## Discussion

BET proteins regulate multiple gene expressions involved in cancer pathogenesis and are emerging therapeutic targets. Results from the first clinical trials confirm the antitumor potential of BETis, but their efficacy as single agents seems to be limited. Instead, combination therapies and the next generation of compounds such as BET degraders may open new possibilities for targeting BET proteins in cancer [25, 26]. Ongoing BETi-based combination therapies include kinase inhibitors, immune modulators, epigenetic drugs, and hormone therapy, and clinical outcomes are awaited. Notably, the expression of several metabolic genes can be epigenetically regulated by BET proteins, and BET protein target genes including *MYC* are master regulators of cellular metabolism. It is thus reasonable to postulate that targeting BET proteins may directly or indirectly affect cancer metabolism, rendering BETi-treated cells dynamically undergo metabolic remodeling in this context. In support of this notion, histone deacetylase (HDAC) inhibition elicits metabolic reprogramming by targeting super-enhancers in glioblastoma, and combination treatment with HDAC and fatty acid oxidation (FAO) inhibitors exhibits a better therapeutic potential [42]. However, how BETi induces metabolic remodeling and whether a metabolic targeting strategy can be combined with BETi for cancer treatment have not been explored. Here using liver cancer as a model, we uncover a GDH1-dependent glutamine metabolic remodeling upon BET inhibition, and provide a proof-of-principle for “synthetic lethality” targeting GDH1-OXPHOS metabolic axis and BET proteins for improved cancer treatment (Fig. 6j).

We find that both BRD2 and BRD4 but not BRD3 are required for liver cancer cell growth, and even transient BET suppression using pan-BETis (JQ-1 and ABBV075) is sufficient to impede cell proliferation and xenograft tumor growth. From a therapeutic perspective, short-term treatment may also reduce the side effects of BET inhibition. To address how BET inhibition may affect liver cancer cell metabolism, we started with RNA-seq and find that BETi decreases expressions of genes in glucose and mitochondrial metabolism, consistent with the notion that BET proteins generally facilitate gene transcriptional activation. Interestingly, GDH1 is identified as one of the few metabolic genes upregulated by BET inhibition. Through the production of α-KG, GDH1 contributes to redox homeostasis in breast and lung cancer cells [37], promotes anoikis resistance in liver kinase B1 (*LKB1*)-deficient lung cancer [43], and is required for glioblastoma cells to survive under glucose limitation [44]. In line with GDH1 upregulation, BETi increases not only the abundance of multiple glutamate metabolism and TCA cycle metabolites but also the activity of glutamine oxidative metabolism, which provides anaplerotic metabolites including α-KG for TCA cycle. As a result, BETi-treated cells are able to maintain energy homeostasis despite impaired growth. Functionally, targeting GDH1, GLS1, or OXPHOS consistently results in synthetic lethality with BETi, while none of these single treatments induce dramatic cell death. Collectively, these findings demonstrate that BETi-treated cancer cells undergo GDH1-mediated glutamine metabolic remodeling to maintain energy homeostasis and viability, and blocking this adaptation process elicits energy crisis and synthetic lethality with BET inhibition.

Context-dependent GDH1 modulation can occur at different levels. For example, the transcription factor pleomorphic adenoma gene 1 (PLAG1) controls GDH1 expression during anoikis resistance [43], the protein but not mRNA level of GDH1 is induced when proliferating mammary epithelial cells undergo quiescence [45], and GDH1 enzymatic activity but not the protein level is elevated when glucose oxidation is inhibited in glioblastoma cells [44]. We have identified a distinct mechanism of GDH1 upregulation by BET inhibition in liver cancer cells, which is through the downregulation of *miR-30a* independent of MYC. We demonstrate that *miR-30a* negatively regulates *GDH1* mRNA stability, and *miR-30a* levels are reduced upon BET inhibition. Although *miR-30a* downregulation may affect multiple gene expressions, our results support that GDH1 is the one involved in glutamine metabolic remodeling upon BET inhibition. It remains unclear how BETi decreases *miR-30a* levels and whether other mechanisms also regulate GDH1 in this context.

Finally, we show that simultaneously targeting BET proteins and GLS1-GDH1-OXPHOS metabolic axis limits liver tumor growth, even in tumors driven by ectopic *MYC* overexpression. In our MYC/p53 model, three-week ABBV075/IACS combination therapy significantly extends the survival of animals compared to monotherapy cohorts. This treatment regimen seems to be well-tolerated, despite that the treated animals initially lose and progressively recover their body weight. It should be noted that a recent clinical trial revealed that IACS monotherapy is associated with toxic side effects including peripheral and optic neuropathy, and hence only modest evidence of enhanced efficacy is observed in patients with acute myeloid leukemia or advanced breast cancer and pancreatic cancer [46]. This observation strongly suggests that it is necessary to identify the cancer type/patients suitable for the treatment or optimize the treatment regimen to reduce the side effects of targeting complex I. Indeed, complex I loss exposes fermentation as a therapeutic target in Hürthle cell carcinoma and has implications for other tumors bearing mutations that irreversibly damage mitochondrial respiration [47, 48]. We herein show that BET inhibition, on the one hand, has a strong negative impact on glycolysis, on the other hand, increases GDH1-mediated glutamine metabolic remodeling, establishing a synthetic lethality to target both pathways. Notably, BETi/OXPHOSi combination therapy is more effective to BETi-responsive liver cancer cells, as non-cancerous cells (IMR90) and BETi-less sensitive SNU449 cancer cells do not respond to this treatment, raising a remaining question about how to stratify liver cancer patients that would benefit from the combination therapy. In addition to OXPHOS inhibition, our findings also suggest that targeting induced metabolic dependency upstream of OXPHOS, such as GLS1 or GDH1, can also induce synthetic lethality in BETi-responsive liver cancer. As a new generation of BETis is emerging, such as BRD4 PROTAC (ARV-825), together with the suitability of GLS1 and GDH1 as drug targets, one can anticipate that targeting BET proteins and GLS1-GDH1 metabolic axis may hold therapeutic potential, which is worth being fully evaluated in the future.

In summary, our study provides mechanistic insights into the metabolic adaptation underlying BET inhibition in liver cancer and proposes an option for synthetic lethality targeting GDH1-dependent glutamine metabolism and BET proteins for improved combination treatment of liver cancer.

## Materials and methods

### Cell culture

Huh7, Hep3B, IMR90, SNU449, and 293T cell lines were purchased from the American Type Culture Collection (ATCC) cell bank. HepaMP9-1 cell line was generated in the Simon lab at the University of Pennsylvania. Huh7, Hep3B, 293T, and HepaMP9-1 cells were cultured in Dulbecco’s modified Eagle’s medium (DMEM) containing 10% fetal bovine serum (FBS) (hereafter growth medium). SNU449 cells were cultured in Roswell Park Memorial Institute (RPMI) medium containing 10% FBS. The cells were routinely tested to exclude mycoplasma contamination. For cell growth assays, single cells in complete medium were seeded in 6-well plates (5 × 10^4^/well, in triplicate), the growth medium was changed every 2 days, and cell numbers were counted with a hemacytometer. For the clonogenicity assay, single cells were seeded in 12-well plates (2 × 10^3^/well, in triplicate) and cultured for 10 − 12 days, and the medium was changed every 3 days. For suspension culture, single cells were seeded on ultra-low attachment culture dishes (Corning) in DMEM/F12 serum-free medium (Invitrogen) [29], which contained 2 mmol/L L-glutamine, 1% sodium pyruvate (Invitrogen), 100 μg/mL penicillin G, and 100 U/mL streptomycin supplemented with 20 ng/mL epithelial growth factor (Invitrogen), 10 ng/mL fibroblast growth factor-2 (Invitrogen), N2 (Invitrogen), and B27 (Invitrogen). The cells were incubated in a CO_2_ incubator for 2 weeks, and the numbers of oncosphere cells were counted under a stereomicroscope (Olympus, Tokyo, Japan).

### Crystal violet staining

Cultured cells were washed once with 1 × PBS and then stained with 1 × PBS solution containing 20% methanol and 0.25% crystal violet for 30 min. The cells were then washed to remove residual crystal violet.

### Chemicals

JQ-1 (#A1910) and CB839 (#B4799) were purchased from APExBIO. ABBV075 (#HY-100015), ARV-825 (#HY16954), and IACS010759 (#HY-112037) were purchased from MedChemExpress. R162 (#E1170) was purchased from Selleck. [U-^13^C]-glutamine and [U-^13^C]-glucose were purchased from Cambridge Isotope Laboratories. Acetonitrile (ACN), methanol (MeOH), and formic acid at liquid chromatography grade were purchased from Thermo Fisher company. Methylene diphosphate was purchased from Sigma.

### Western blot analysis

Cells were washed with 1 × PBS and lysed using lysis buffer (150 mmol/L NaCl, 10 mmol/L Tris pH 7.6, 0.1% SDS, and 5 mmol/L EDTA) containing Halt Protease and Phosphatase Inhibitor Cocktail (Thermo Fisher Scientific, 78445). Samples were centrifuged at 12,000 rpm for 20 min at 4°C. Protein lysates were separated by SDS–PAGE and transferred to PVDF membranes (Millipore). All membranes were incubated with indicated primary antibodies diluted in TBST (20 mmol/L Tris pH 7.5, 150 mmol/L NaCl, and 0.1% Tween-20) with 5% bovine serum albumin (Sigma–Aldrich, A7906) overnight at 4°C. After TBST washes, membranes were incubated with a secondary antibody and developed with Western Lightning Plus-ECL, Enhanced Chemiluminescence Substrate (PerkinElmer, cat. NEL103E001EA). The following primary antibodies were used: Cell Signaling Technology: phospho-AMPKα (Thr172) (40H9) Rabbit mAb (#2535) and AMPKα (#2532); Abclonal: HSP90 (#A5027), HK2 (#A0994), GLUT1 (#A11208), LDHA (#A0861), GDH1(#A5176), and GLS (#A11043); Proteintech: BRD4 (#67374), MYC (#67447-1-Ig), GOT1 (#14886-1-AP), GOT2 (#14800-1-AP), GPT1 (#16897-1-AP), GPT2 (#16757-1-AP), PSAT1 (#10501-1-AP), and TFAM (#23996-1-AP); Santa Cruz: BRD2 (#sc-514103) and BRD3 (#sc-81202).

### Reverse transcription and qPCR

Total RNA was isolated using RNeasy Mini Kit (Qiagen, Cat. 74104). cDNA was synthesized using a High-Capacity RNA-to-cDNA kit (Vazyme, Cat. R323-01). qPCR was performed using ViiA7 Real-Time PCR system (Applied Biosystems) with SYBR green master mix (Vazyme, Cat. Q711-02). Relative mRNA levels were normalized to 18S ribosomal RNA. qPCR primers for miRNAs are summarized in Supplementary Table S1. Other qPCR primers are summarized in Supplementary Table S2.

### PI/Annexin V staining and flow cytometry

Cell death was determined using the FITC–Annexin V, PI Kit (#556547) from BD Biosciences according to the manufacturer’s instructions. Flow cytometry was performed using the BD FACS Calibur instrument (BD FACS Calibur), with dead cells represented as Annexin V-positive population, and resultant data were analyzed with the FlowJo 10.6.2 software.

### Metabolomics

Huh7 cells were treated with vehicle or JQ-1 (0.5 μmol/L) for 48 h, and then replated in 10-cm plates overnight. For metabolite extraction, the medium was removed by aspiration, and the metabolism was immediately quenched (without any washing steps) by adding 500 μL 40:40:20 acetonitrile:methanol:water containing 0.5% formic acid (−20°C). After 5−10 s, the cell extract was neutralized with 44 μL 15% ammonium bicarbonate. The cell extracts were transferred to 1.5-mL tubes and stored at −80°C overnight and further cleared by centrifuging at 15,000 × g for 10 min to remove proteins. The supernatant was used for LC–MS analysis. Targeted metabolomics analysis was performed using the Shimadzu Prominence HPLC system (ExionLC AD) interfaced with a QTRAP 6500+ system (AB SCIEX). The sample injection volume was 5 µL. Metabolites were separated through an iHILIC-(P) Classic HILIC column (100 mm × 4.6 mm with 3.5 μm particle size, Waters) with column temperature maintained at 40°C. The mobile phase consisted of 20 mmol/L ammonium acetate in 25 mmol/L ammonia water (mobile phase A) and acetonitrile (mobile phase B) and was run at a flow rate of 0.4 mL/min. The gradient was as follows: 0 min, 85% B; 0.1 min, 85% B; 3.5 min, 32% B; 12 min, 2% B; 16.5 min, 2% B; 17 min, 85% B; 26 min, 85% B. The mass spectrometer was run in multiple reaction monitoring mode. The electrospray ionization (ESI) source parameters were as follows: source temperature of 475°C, the ion source gas 1 and 2 at 60 psi, the curtain gas at 35 psi, and the ion spray voltage at 4850 V or −4500 V for positive or negative modes, respectively. Data were processed and analyzed using SCIEX OS software.

### [U-^13^C]-glucose and [U-^13^C]-glutamine labeling and LC–MS

All ^13^C studies were performed in DMEM medium containing 10% dialyzed FBS and prepared so that 100% of either the glucose or glutamine pool was labeled with ^13^C, and the other pool was unlabeled. Huh-7 cells were treated with vehicle or JQ-1 (0.5 μmol/L) for 48 h, and replated in 6-well plates (10^6^/well) overnight. Then the cells were switched to a fresh culture medium so that cells should reach 60%−90% confluence the next day. At the beginning of the experiment, cells were switched into labeled [U-^13^C]-glucose or [U-^13^C]-glutamine DMEM medium containing 10% dialyzed FBS and incubated for 4 h. The metabolite extraction and LC–MS sample preparation steps were identical to the targeted metabolomics experiment. Isotope labeling data acquisition was performed using a Shimadzu LC system coupled to a TripleTOF mass spectrometer (QTOF 6600+, ABSciex, made in Woodlands Central Industrial Estate, Singapore). Chromatographic separation was achieved using a HILIC column (IHILIC-(P) Classic column, 5 μm, 150 mm × 2.1 mm, 200 A, made in Sweden). The mobile phase A was 20 mmol/L ammonium acetate, 0.1% ammonium hydroxide, and 2.5 μmol/L methylene diphosphate in 95:5 water:ACN, and mobile phase B was ACN. The gradient was as follows: 0 min, 85% B; 2 min, 85% B; 7 min, 65% B; 12 min, 35% B; 12.1 min, 20% B; 15.9 min, 20% B; 16 min, 85% B; 23 min, 85% B. The flow rate was set at 0.2 mL/min with a sample injection volume of 5 µL and the total run time at 23 min. ESI parameters setup was GS1, 60; GS2, 60; CUR, 35; temperature, 500; ISVF, −4500 in negative modes. For each metabolite, the areas of ^12^C and various ^13^C isotopologue peaks were integrated using El-MAVEN software (version 0.12.1). Correction for natural isotope abundance was performed using R package AccuCor. The labeling fraction of a specific isotopologue was calculated as the area of that isotopologue divided by the sum of all isotopologues’ area [49].

### Constructs and cell transfections

shRNAs were cloned into pLko.1-puro (Addgene #8453) linearized with AgeI and EcoRI. sgRNAs were cloned into pLentiCRISPR v2 Lko.1-puro (Addgene #52961) linearized with BsmBI. The following oligonucleotides for shRNAs were used,

*shBRD2*: CCCTGCCTACAGGTTATGATT;

*shBRD3*: GCTGATGTTCTCGAATTGCTA;

*shBRD4*: CCTGGAGATGACATAGTCTTA;

*shGDH1-1*: GCCATTGAGAAAGTCTTCAAA;

*shGDH1-2*: GCAGAGTTCCAAGACAGGATA;

*shMYC*: CCTGAGACAGATCAGCAACAA.

The following oligonucleotides for sgRNAs were used,

*sgBRD4-1*: TAAGATCATTAAAACGCCTA;

*sgBRD4-2*: TCTTCCTCCGACTCATACGT.

To produce lentiviruses, 293T cells were co-transfected with LentiCRISPR v2 plasmids along with packaging plasmids psPAX2 and pMD2.G using PEI transfection reagent (#23966-100, Polysciences). Lentiviruses were collected 48 h after transfection. *miR-30a* inhibitor was purchased from GenePharma and transfected using Lipofectamine™ RNAiMAX Transfection Reagent (ThermoFisher Scientific, #13778075) following the manufacturer’s instructions. For luciferase reporter assays, *GDH1* 3’-UTR was amplified using the following primers,

Forward: ATCGCTCGAGTGATGAAAGCTGCGCACTAGTTCTGCAGACCTATCACAAGT;

Reverse: ATCGAAGCTTAAGACTATGCTTTCAGGGAT,

and cloned into pMIR-REPORT™ miRNA expression reporter vector. Mutant 3’-UTR reporter was generated through site-directed mutagenesis and confirmed by DNA sequencing. Luciferase activity was quantified using a dual luciferase assay kit (Promega). Relative luciferase activity was calculated by normalizing relative luciferase activities to *Renilla* activities in each well.

### Animal experiments

All mouse experiments were reviewed and approved by the Institutional Animal Care and Use Committee at Fudan University. Male C57BL/6 and female NCG mice were purchased from GemPharmatech Co., Ltd (Nanjing, China), and were maintained in a specific-pathogen-free animal facility at Fudan University. For subcutaneous injection, 5 × 10^6^ Huh7 cells mixed with Matrigel (BD Biosciences, #356234) (with a ratio of 1:1) in a final volume of 100 μL were injected into the blanks of NCG mice, respectively. The tumor volume was monitored by caliper measurements. When tumor volume reached 100 mm^3^, NCG mice were randomized and divided into groups for drug treatment. For HDTI, vectors were prepared using the EndoFree-Maxi Kit (Qiagen) and resuspended in a sterile 0.9% NaCl solution/plasmid mixture containing 10 μg pT3-MYC (Addgene #92046), 10 μg pX330-p53 (Addgene 59910), and 2.5 μg CMV-SB13 transposase. A total volume mixture corresponding to 10% of body weight was injected via lateral tail vein in 5–8 s into 6-week-old male C57Bl/6 mice, as previously. For drug treatment, ABBV075 and IACS were diluted in 0.5% methyl cellulose solution (MC), and administrated via oral gavage once daily at 1 mg/kg and 7.5 mg/kg body weight, respectively. 0.5% MC was used as vehicle. The NCG mice were treated for 12 days, and HDTI-treated C57BL/6 mice were treated for 3 weeks. After treatment, the mice were carefully monitored and euthanized when moribund, and tumor burden was confirmed. For hematoxylin-eosin (HE) staining, mouse tissues were fixed in 4% paraformaldehyde immediately after collection, dehydrated, and stained with HE as previously [17].

### RNA-seq and GSEA

Total RNA was extracted from the vehicle and BETi-treated Huh7 cells using a RNeasy mini kit (Qiagen). The RNA quality test, library construction, and sequencing were performed by Novogene Corporation (Beijing). For data analysis, FASTQ files were checked for quality using FastQC and Qualimap. Alignment was performed using the STAR aligner under default settings with the hg19 reference genome. Raw counts of gene transcripts were obtained from the resulting bam files using the feature counts. The raw count matrix was subsequently imported into R-studio (R version 3.3.3) and used as input for DESeq2 following the vignette of the package for normalization and differential gene expression analysis. Salmon/Sailfish was used in parallel to normalize and quantitate gene expression in transcripts per million through quasi-alignment. GSEA was run for the contrast in pre-ranked mode using the DESeq2 statistic as the ranking metric. When there were redundant mappings, the statistic with the highest absolute value was chosen [50].

### Statistical analysis

All results were obtained from three independent biological experiments, using three technical replicates per condition, unless stated otherwise. Statistical analyses per experiment are indicated in figure legends. Statistical tests were performed in GraphPad Prism 9.0.0 using Student’s two-tailed unpaired *t*-test for pairwise comparisons, one-way analysis of variance (ANOVA) for multiple comparisons, two-way ANOVA for multiple comparisons involving two independent variables, or log-rank test for comparisons of survival distributions of two groups. Statistical data are presented as mean ± SEM of at least three independent experiments. A *P* value < 0.05 was considered significant.

### Data and code availability

TCGA liver cancer dataset was downloaded and analyzed at the Molecular Profiling Facility at the University of Pennsylvania as previously. All sequencing data have been deposited in the Gene Expression Omnibus under the series GSE184065. The software and algorithms for data analyses used in this study were all well-established from previous work and were referenced throughout the manuscript. No custom code was used in this study. All data supporting the findings of this study are available from the corresponding author upon reasonable request.

## Supplementary Material

loae016_suppl_Supplementary_Figures_S1-S6

loae016_suppl_Supplementary_Tables_S1-S2
