# Supplementary material for: BET inhibition induces GDH1-dependent glutamine metabolic remodeling and vulnerability in liver cancer
Source: Life Metab. 2024 Apr 26;3(4):loae016. doi: 10.1093/lifemeta/loae016 (PMC11749653; doi:10.1093/lifemeta/loae016)
Supplement: loae016_suppl_Supplementary_Figures_S1-S6 [file loae016_suppl_Supplementary_Figures_S1-S6.docx]

**
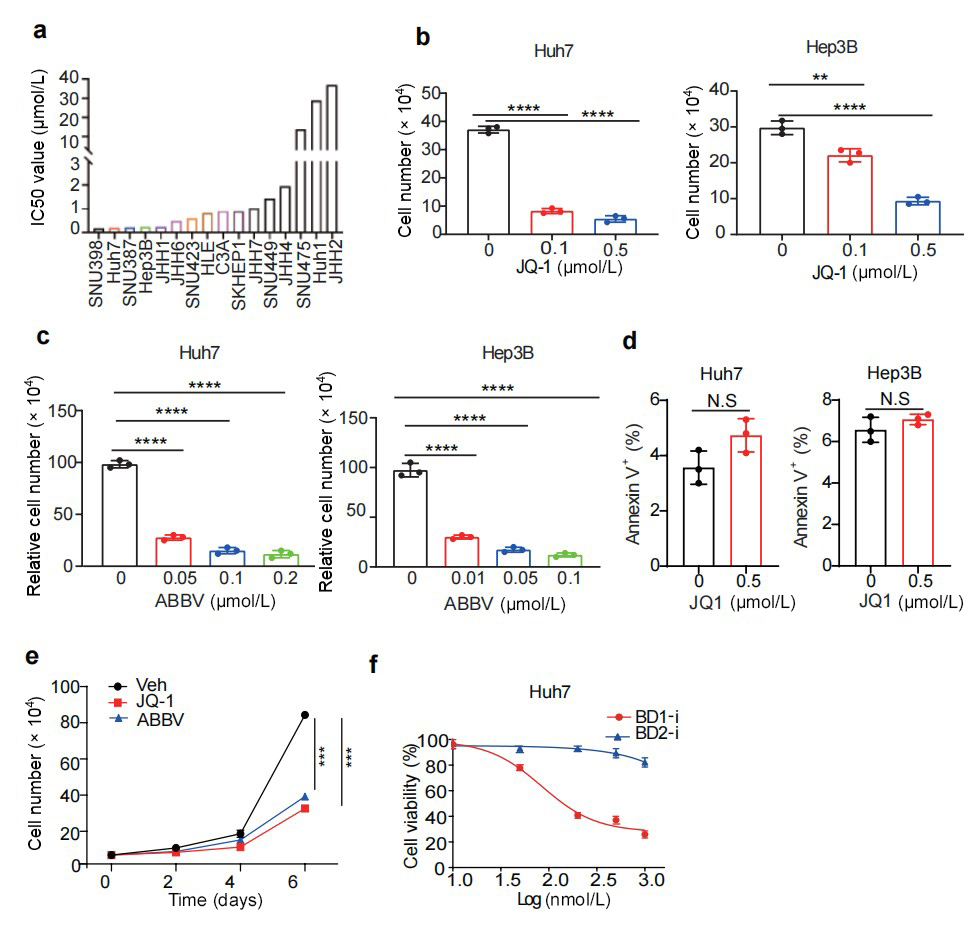
**

**Supplementary Figure S1** BET inhibition blunts HCC cell growth and induces differentiation. (a) JQ-1 IC50 (half maximal inhibitory concentration) values across liver cancer cell lines from the “The Genomics of Drug Sensitivity in Cancer Project”. (b) Quantification of Huh7 and Hep3B cell numbers after JQ-1 treatment for 48 h. (c) Quantification of Huh7 and Hep3B cell numbers after ABBV075 treatment for 48 h. (d) Statistic analysis of the percentage of Annexin V^+^ Huh7 and Hep3B cells from the vehicle and JQ-1 treatment groups. € Cell growth assay for replated Huh7 cells after vehicle, JQ-1, or ABBV075 treatment for 48 h. (f) Relative cell viability in Huh7 cells treated with indicated doses of BD1 and BD2 inhibitors. All statistical graphs show the mean ± SEM. *P* values were calculated using one-way ANOVA. All experiments were performed in biological triplicate. ^**^*P* < 0.01, ^***^*P* < 0.001, ^****^*P* < 0.0001.


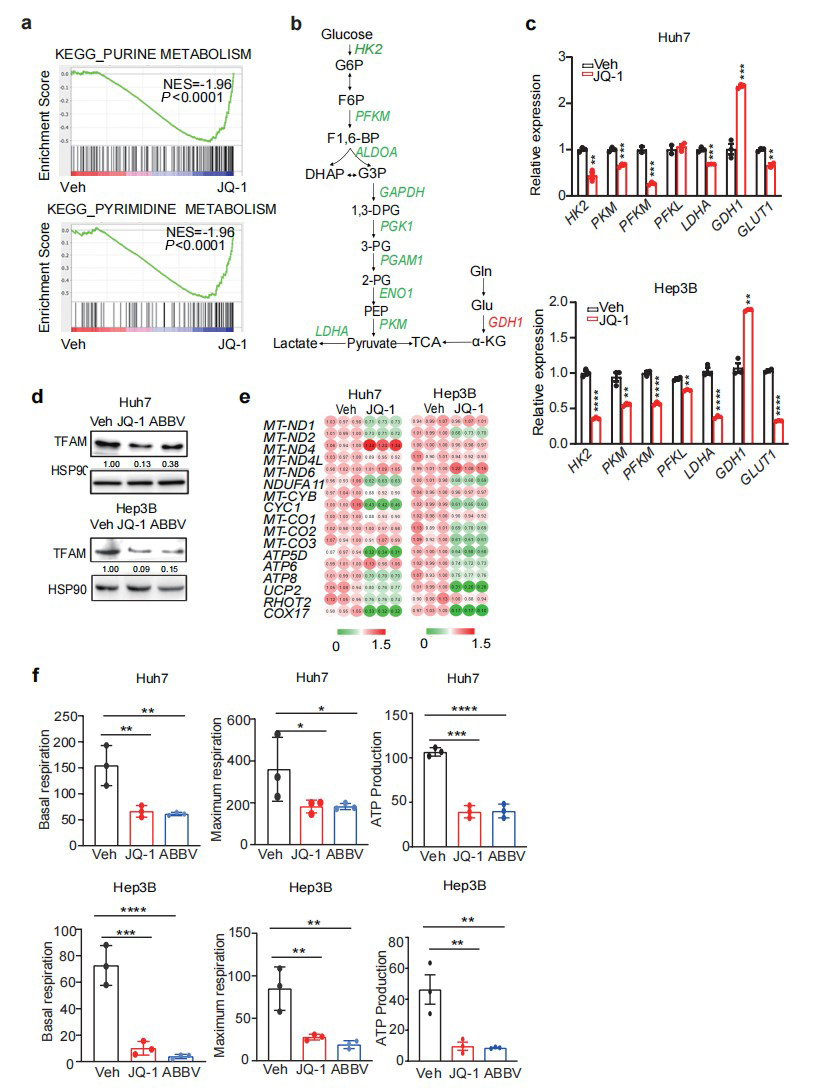


**Supplementary Figure S2** Metabolic gene expression changes in liver cancer cells upon BET inhibition. (a) GSEA for purine and pyrimidine metabolism from the vehicle and JQ-1 groups based on RNAsequencing (RNA-seq) data. (b) A scheme of glycolytic gene expression change upon JQ-1 treatment of Huh7 cells. (c) qPCR analysis of glycolysis gene expression in JQ-1-treated Huh7 and Hep3B cells. (d) Western blot analysis of TFAM from the vehicle and BETi-treated Huh7 and Hep3B cell lysates. HSP90 is used as a loading control. (e) qPCR analysis of mitochondrial ETC subunit gene expression in the vehicle and JQ-1-treated Huh7 and Hep3B cells. (f) Quantification of seahorse assay in the vehicle and BETi-treated Huh7 and Hep3B cells. All statistical graphs show the mean ± SEM. *P* values were calculated using a two-tailed Student’s *t*-test (c) and one-way ANOVA (f). All experiments were performed in biological triplicate. ^*^*P* < 0.05, ^**^*P* < 0.01, ^***^*P* < 0.001, ^****^*P* < 0.0001.


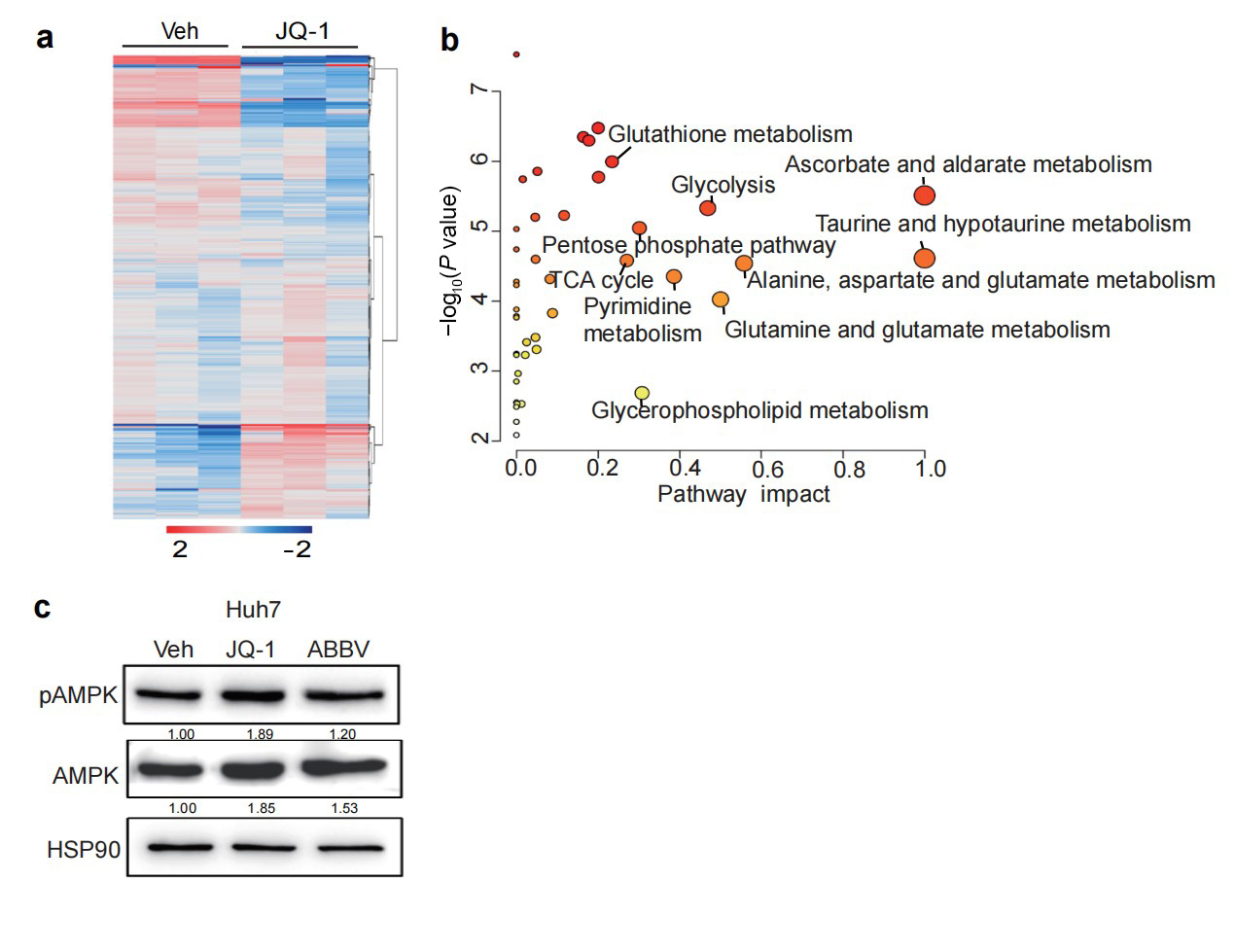


**Supplementary Figure S3**  Metabolomic profiling of liver cancer cells upon BET inhibition. (a) A heatmap of untargeted metabolomics in the vehicle and JQ-1 treated Huh7 cells. (b) Metabolic pathway analysis based on untargeted metabolomics in a. (c) Western blot analysis of AMPK and pAMPK levels in the vehicle and BETi-treated Huh7 cell lysates. HSP90 is used as a loading control. All statistical graphs show the mean ± SEM. *P* values were calculated using a two-tailed Student’s *t*-test. All experiments were performed in biological triplicate.


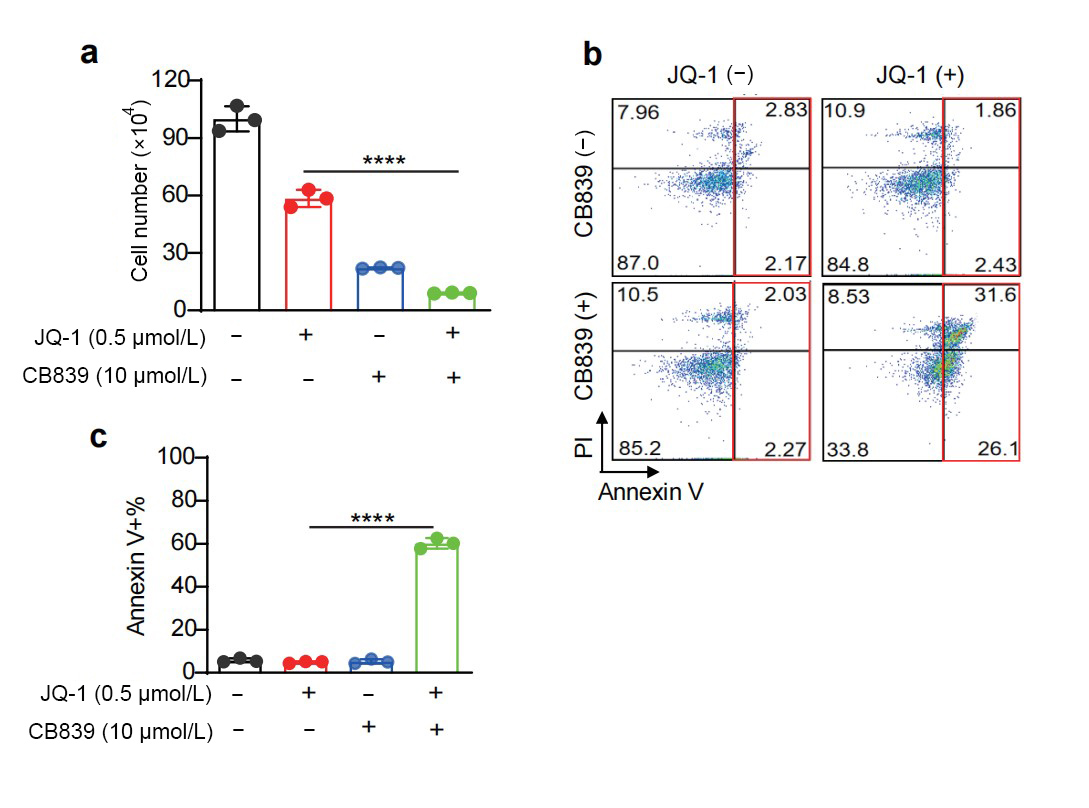


**Supplementary Figure S4** Targeting GLS1 sensitizes liver cancer cells to BET inhibition. (a) Quantification of cell numbers for Huh7 cells treated with JQ-1 and/or CB839. (b) Representative flow cytometry plot of PI and annexin V staining of Huh7 cells from indicated groups. (c) Statistic analysis of the percentage of annexin V^+^ Huh7 cells in the indicated treatment groups. All statistical graphs show the mean ± SEM. *P* values were calculated using one-way ANOVA. All experiments were performed in biological triplicate. ^****^*P* < 0.0001.


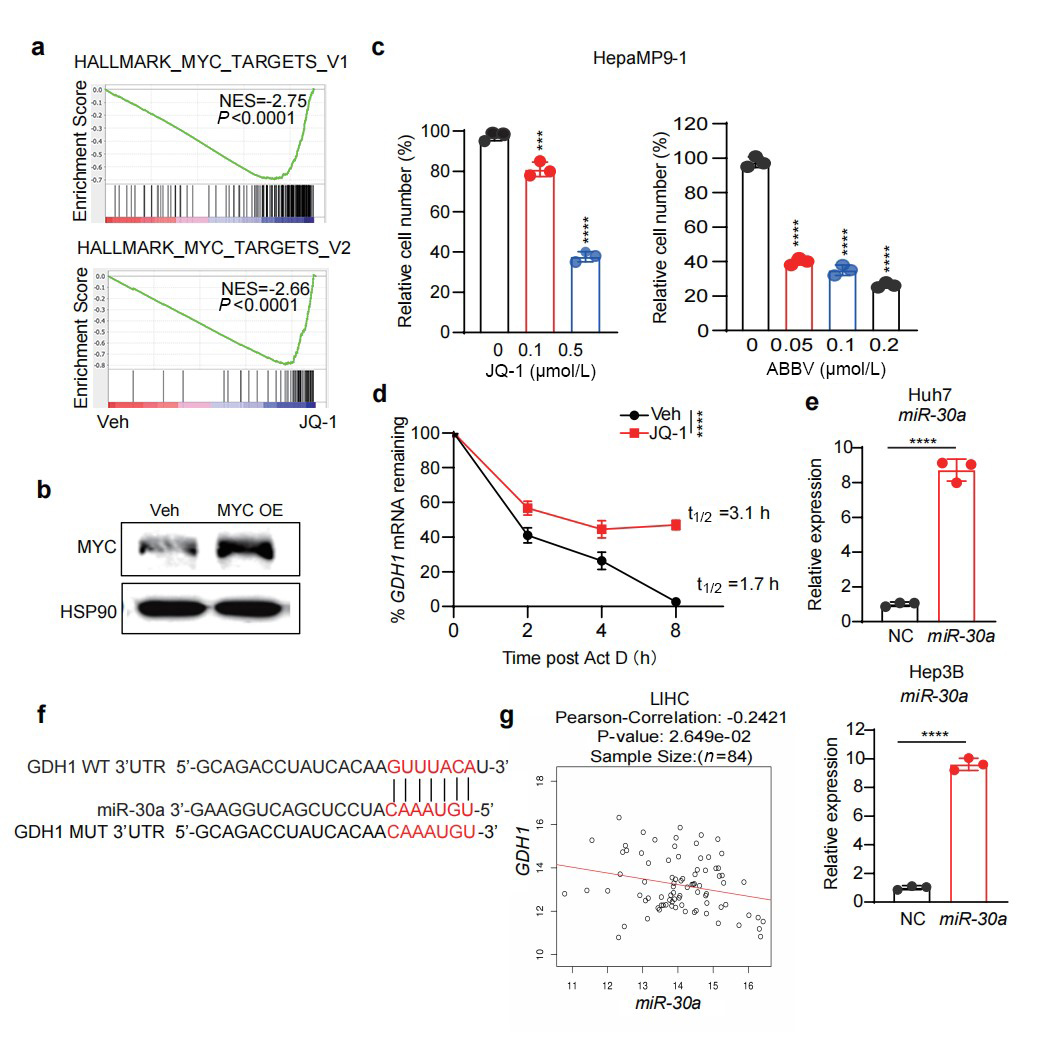


**Supplementary Figure S5** MYC-independent GDH1 regulation by BET inhibition. (a) GSEA for MYC targets from the vehicle and JQ-1 groups based on RNA-seq data. (b) Western blot analysis of control and *MYC*-overexpressed Huh7 cells. (c) Relative cell numbers of HepaMP9-1 cells treated with BETi for 48 h. (d) qPCR analysis of *GDH1* mRNA stability in the vehicle and JQ-1 treated Huh7 cells. €(e) qPCR analysis of *miR30a* levels in control and *miR30a*-overexpressed Huh7 and Hep3B cells. (f) Identification of GDH1 as a target by TargetScan prediction. (g) Correlation of *miR30a* and *GDH1* transcript levels in human HCC samples. All statistical graphs show the mean ± SEM. *P* values were calculated using one-way ANOVA (a) and a two-tailed Student’s *t*-test (d and e). All experiments were performed in biological triplicate. ^***^*P* < 0.001, ^****^*P* < 0.0001.


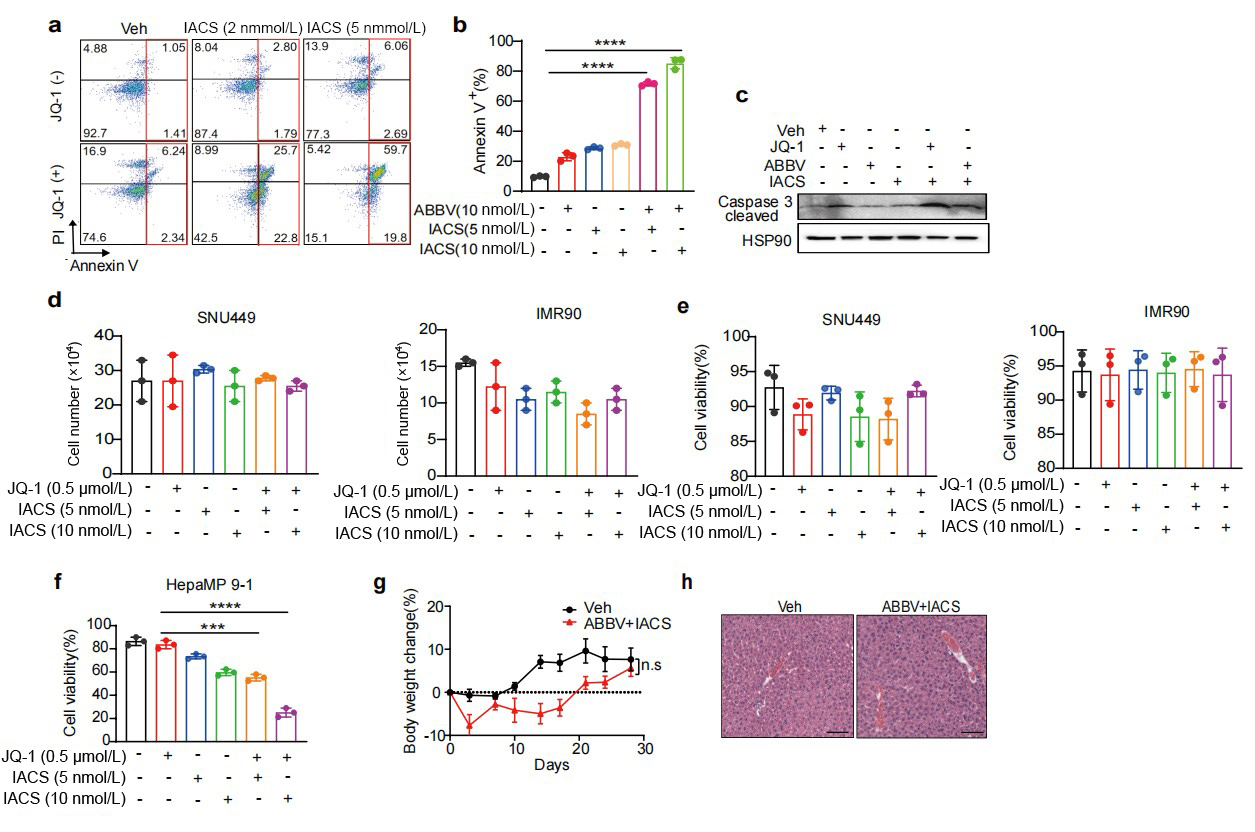


**Supplementary Figure S6**  Combined BET and OXPHOS inhibition limit liver tumor growth *in vitro* and *in vivo*. (a) Representative flow plot of PI/Annexin V staining in the indicated JQ1 and IACS treatment groups for 6 days. (b) Quantification of the percentage of Annexin V^+^ Huh7 cells treated with indicated ABBV075 and IACS combination for 6 days. (c) Western blot analysis of cleaved caspase 3 in Huh7 cells from indicated treatment groups. (d) Quantification of the numbers of SNU449 and IMR90 cells treated with indicated drug combinations for 6 days. (e) Quantification of the viability (% Annexin V^−^) of SNU449 and IMR90 cells treated with indicated drug combinations for 6 days. (f) Quantification of the viability (% Annexin V^−^) of HepaMP9-1 cells treated with indicated drug combinations for 6 days. (g) Body weight change over time in the vehicle and combination treatment cohorts. (h) Representative HE staining of liver sections from the vehicle and combination treatment cohorts. Scale bar: 100 µm. All experiments were performed in biological triplicate. *P* values were calculated using one-way ANOVA. ^***^*P* < 0.001, ^****^*P* < 0.0001.
