# Supplementary material for: BET inhibition induces GDH1-dependent glutamine metabolic remodeling and vulnerability in liver cancer
Source: Life Metab. 2024 Apr 26;3(4):loae016. doi: 10.1093/lifemeta/loae016 (PMC11749653; doi:10.1093/lifemeta/loae016)
Supplement: loae016_suppl_Supplementary_Tables_S1-S2 [file loae016_suppl_Supplementary_Tables_S1-S2.docx]

**Supplementary Table S1** Primer sequences for miRNA reverse transcription (RT) and qPCR analysis.

| Gene | RT primer sequence (5’−3’) | qPCR Primer sequence (5’−3’) |
| --- | --- | --- |
| *miR-3163* | GTCGTATCCAGTGCAGGGTCCGAGGTATTCGCACTGGATACGACGTCTTA | F: GCCGAGTATAAAATGAGGGCAG  R: AGTGCAGGGTCCGAGGTATT |
| *miR-30a-5p* | GTCGTATCCAGTGCAGGGTCCGAGGTATTCGCACTGGATACGACCTTCCA | F: GCCGAGTGTAAACATCCTCGAC  R: AGTGCAGGGTCCGAGGTATT |
| *miR-30b-5p* | GTCGTATCCAGTGCAGGGTCCGAGGTATTCGCACTGGATACGACAGCTGA | F: GCCGAGTGTAAACATCCTACAC  R: AGTGCAGGGTCCGAGGTATT |
| *miR-30c-5p* | GTCGTATCCAGTGCAGGGTCCGAGGTATTCGCACTGGATACGACGCTGAG | F: GCCGAGTGTAAACATCCTACACT  R: AGTGCAGGGTCCGAGGTATT |
| *miR-30d-5p* | GTCGTATCCAGTGCAGGGTCCGAGGTATTCGCACTGGATACGACCTTCCA | F: GCCGAGTGTAAACATCCCCGAC  R: AGTGCAGGGTCCGAGGTATT |
| *miR-30e-5p* | GTCGTATCCAGTGCAGGGTCCGAGGTATTCGCACTGGATACGACCTTCCA | F: GCCGAGTGTAAACATCCTTGAC  R: AGTGCAGGGTCCGAGGTATT |
| *U6* | AACGCTTCACGAATTTGCGT | F: CTCGCTTCGGCAGCACA  R: AACGCTTCACGAATTTGCGT |
| F, forward; R, reverse. | | |

**Supplementary Table S2** Primer sequences used for qPCR analysis.

| Gene | qPCR Primer sequence (5’−3’) |
| --- | --- |
| *PSAT1* | F: ACTTCCTGTCCAAGCCAGTGGA  R: CTGCACCTTGTATTCCAGGACC |
| *GPT2* | F: ATCCTCACGCTGGAGTCCATGA  R: ATGTTGGCTCGGATGACCTCTG |
| *GOT1* | F: GGACCTGGAACCACATCACTGA  R: ACCACTTGGCAGCAGGTAGATG |
| *GOT2* | F: CCAAGGCTTTGCCAGTGGTGAT  R: AGTGAAGGCTCCTACACGCTCA |
| *GLS* | F: CAGAAGGCACAGACATGGTTGG  R: GGCAGAAACCACCATTAGCCAG |
| *GDH1* | F: CTCCAGACATGAGCACAGGTGA  R: CCAGTAGCAGAGATGCGTCCAT |
| *HK2* | F: GAGTTTGACCTGGATGTGGTTGC  R: CCTCCATGTAGCAGGCATTGCT |
| *PKM* | F: ATGGCTGACACATTCCTGGAGC  R: CCTTCAACGTCTCCACTGATCG |
| *PFKM* | F: GCTTCTAGCTCATGTCAGACCC  R: CCAATCCTCACAGTGGAGCGAA |
| *PFKL* | F: AAGAAGTAGGCTGGCACGACGT  R: GCGGATGTTCTCCACAATGGAC |
| *LDHA* | F: GGATCTCCAACATGGCAGCCTT  R: AGACGGCTTTCTCCCTCTTGCT |
| *GLUT1* | F: TTGCAGGCTTCTCCAACTGGAC  R: CAGAACCAGGAGCACAGTGAAG |
| *MT-ND1* | F: GGCTATATACAACTACGCAAAGGC  R: GGTAGATGTGGCGGGTTTTAGG |
| *MT-ND2* | F: CTTCTGAGTCCCAGAGGTTACC  R: GAGAGTGAGGAGAAGGCTTACG |
| *MT-ND4* | F: CCCTCGTAGTAACAGCCATTCTC  R: CGACTGTGAGTGCGTTCGTAGT |
| *MT-ND4L* | F: CCCTCGTAGTAACAGCCATTCTC  R: CGACTGTGAGTGCGTTCGTAGT |
| *MT-ND6* | F: GCGATGGCTATTGAGGAGTATCC  R: CACAGCACCAATCCTACCTCCA |
| *NDUFA11* | F: CCTTCCTTGAAGGAGTGGCTAAG  R: AGCCACCGAGGAAGTAGTTCAG |
| *MT-CYB* | F: CCATCTTTGTGGCTGTGCTTGC  R: ACCAGTAGTGCCTCGGTCATCA |
| *CYC1* | F: CCAGATAGCCAAGGATGTGTGC  R: GACTGACCACTTGTGCCGCTTT |
| *MT-CO1* | F: GATGAGCAGCTTTTCCAGACGAC  R: AACTGGACACCGAACAGCAGCT |
| *MT-CO2* | F: CGGTGAAACTCTGGCTAGACAG  R: GCAAACCGTAGATGCTCAGGGA |
| *MT-CO3* | F: GATGAGCAGCTTTTCCAGACGAC  R: AACTGGACACCGAACAGCAGCT |
| *ATP-5D* | F: CGGAGCCTTCGGCATCCTGG  R: AAGAGTCGGCGTTCACTGCGAT |
| *ATP6* | F: GTCTGCCTCTTCCTGAACTTGG  R: CCGTCAGTATGACCAGCACATG |
| *ATP8* | F: CACAGATGGCAGTGTCCCAGAA  R: CAGGTACTGCTTGTCCACAGATG |
| *UCP2* | F: TGGTCGGAGATACCAAAGCACC  R: GCTCAGCACAGTTGACAATGGC |
| *RHOT2* | F: GCAGGTCAATGGACAGGAGAAG  R: TGTAGACGCTGGCACAATGTGC |
| *COX17* | F: TTGCCCGGAGACCAAGAAGGC  R: ATTATTTATTCACACAGCAGACCAC |
| F, forward; R, reverse. | |
